# LL37 complexed to double-stranded RNA induces RIG-I-like receptor signalling and Gasdermin E activation facilitating IL-36γ release from keratinocytes

**DOI:** 10.1038/s41419-025-07537-9

**Published:** 2025-03-22

**Authors:** Jennifer Keller, Judit Danis, Isabella Krehl, Eleftheria Girousi, Takashi K. Satoh, Barbara Meier-Schiesser, Lajos Kemény, Márta Széll, W. Wei-Lynn Wong, Steve Pascolo, Lars E. French, Thomas M. Kündig, Mark Mellett

**Affiliations:** 1 Department of Dermatology, University Hospital Zürich (USZ), University of Zürich (UZH), Raemistrasse 100, 8091, Zürich, Switzerland; 2 Department of Immunology, University of Szeged, Szeged, Hungary; 3 HUN-REN-SZTE Dermatological Research Group, University of Szeged, Szeged, Hungary; 4 Department of Dermatology and Allergy, University Hospital, LMU Münich, Germany; 5 Department of Dermatology and Allergology, University of Szeged, Szeged, Hungary; 6 HCEMM-USZ Skin Research Group, University of Szeged, Szeged, Hungary; 7 Department of Medical Genetics, University of Szeged, Szeged, Hungary; 8 HUN-REN-SZTE Functional Clinical Genetics Research Group, University of Szeged, Szeged, Hungary; 9 Department of Molecular Life Sciences, University of Zürich, Winterthurerstrasse 190, 8057, Zürich, Switzerland; 10 Dr. Phillip Frost Department of Dermatology & Cutaneous Surgery, University of Miami, Miller School of Medicine, Miami, USA

**Keywords:** RIG-I-like receptors, Psoriasis

## Abstract

The Interleukin-36 (IL-36) cytokine family have emerged as important players in mounting an inflammatory response at epithelial barriers and tailoring appropriate adaptive immune responses. As members of the Interleukin-1 superfamily, IL-36 cytokines lack a signal peptide for conventional secretion and require extracellular proteolysis to generate bioactive cytokines. Although the IL-36 family plays an important role in the pathogenesis of plaque and pustular psoriasis, little is known about the release mechanisms of these cytokines from keratinocytes and the physiological stimuli involved. Nucleic acid released from damaged or dying keratinocytes initiates early inflammatory signals that result in the breaking of tolerance associated with psoriasis pathogenesis onset. Cathelicidin peptide, LL37 binds to DNA or double-stranded RNA (dsRNA) and activates a type I Interferon responses in plasmacytoid dendritic cells and keratinocytes. Here, we demonstrate that LL37 binds to dsRNA and induces IL-36γ release from human primary keratinocytes. LL37/dsRNA complexes activate RIG-I-like Receptor signalling, resulting in Caspase-3 and Gasdermin E (GSDME) cleavage. Subsequent GSDME pore formation facilitates IL-36γ release. This response is magnified by priming with psoriasis-associated cytokines, IL-17A and IFNγ. IL-36γ release in this manner is largely independent of cell death in primary keratinocytes and lacked extracellular proteolysis of IL-36γ. Conversely, transfection of keratinocytes directly with dsRNA synthetic analogue, Poly(I:C) induces NLRP1 inflammasome activation, which facilitates IL-36γ expression and release in a GSDMD-dependent manner. Inflammasome-associated cell death also enables extracellular processing of IL-36γ by the release of keratinocyte-derived proteases. These data highlight the distinct responses triggered by dsRNA sensors in keratinocytes. Depending on the inflammatory context and magnitude of the exogenous threat, keratinocytes will release IL-36γ coupled with cell death and extracellular cleavage or release the inactive pro-form, which requires subsequent processing by neutrophil proteases to unleash full biological activity, as occurring in psoriatic skin.

**Figure:**
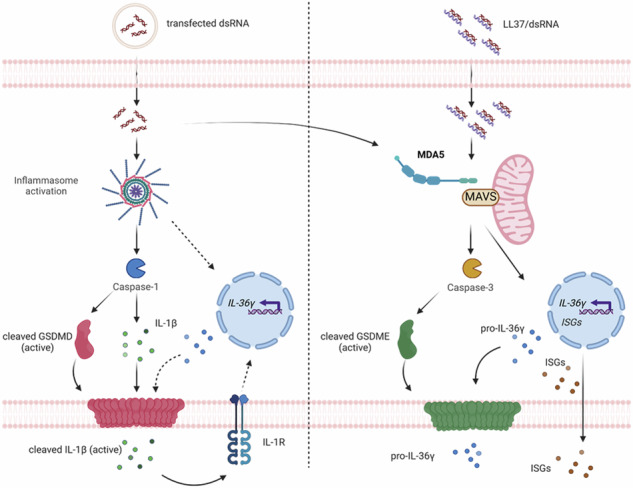
**Cytoplasmic sensing of dsRNA in keratinocytes mediates IL-36γ release via caspase activity and GSDM pore formation** Keratinocytes release IL-36γ upon stimulation with intracellular dsRNA alone or complexed to the psoriasis-associated cathelicidin anti-microbial peptide LL37. Left: Transfected dsRNA triggers NLRP1 inflammasome assembly and IL-1β release, which can enhance IL-36γ expression, resulting in IL-36γ release and extracellular cleavage by released proteases. Right: LL37/dsRNA complexes activate a MDA5-MAVS pathway facilitating the release of IL-36γ through Caspase-3 activation and GSDME pore formation.

**Cytoplasmic sensing of dsRNA in keratinocytes mediates IL-36γ release via caspase activity and GSDM pore formation** Keratinocytes release IL-36γ upon stimulation with intracellular dsRNA alone or complexed to the psoriasis-associated cathelicidin anti-microbial peptide LL37. Left: Transfected dsRNA triggers NLRP1 inflammasome assembly and IL-1β release, which can enhance IL-36γ expression, resulting in IL-36γ release and extracellular cleavage by released proteases. Right: LL37/dsRNA complexes activate a MDA5-MAVS pathway facilitating the release of IL-36γ through Caspase-3 activation and GSDME pore formation.

## Introduction

Interleukin-36 (IL-36) cytokines are an important subfamily of the IL-1 superfamily that mount an inflammatory response at epithelial barriers, acting as an early defence mechanism against infection in the skin, intestine and lung tissue [1–4]. Similar to IL-1, IL-36 cytokines lack a signal peptide for conventional secretion and additionally require proteolytic cleavage of a pro-domain for full biological activity [5]. Furthermore, IL-36 cytokines lack a Caspase-1 cleavage site and are processed extracellularly by bacterial, allergen-associated and neutrophil-derived proteases [5–9]. Cathepsin S, also from keratinocytes can additionally cleave IL-36γ [10]. Infiltration of neutrophils to epidermal tissue in psoriatic skin disease therefore promotes IL-36 cytokine biological activity. Unlike, IL-1β that is constitutively expressed in human keratinocytes [11], IL-36 cytokines are expressed at low levels but are strongly upregulated in response to infection and psoriasis-associated cytokines, including IL-17A [8, 12]. The pro-inflammatory molecules, IL-36α, IL-36β and IL-36γ are significantly upregulated in lesional skin of Psoriasis vulgaris (PsV) and are blocked at the receptor level by the IL-36 Receptor antagonist (IL-36Ra) [13–16]. Loss-of-function mutations in the gene encoding IL-36Ra are associated with Generalised Pustular Psoriasis (GPP), where IL-36 cytokines drive disease pathogenesis by mobilising neutrophil infiltration to epidermal tissue [17, 18]. Mice lacking the IL-36 receptor antagonist do not develop spontaneous psoriasiform disease, but display exacerbated skin inflammation in response to imiquimod, while *IL-36r*- knockout mice are protected against imiquimod-induced disease development [19, 20]. Intradermal injection of recombinant IL-36α to the ears of mice, or IL-36α expression under the control of the Keratin-14 promotor causes a transient inflammatory skin phenotype, which is exacerbated in mice lacking IL-36Ra [21, 22].

Keratinocytes are a major source of IL-36 cytokines in inflammatory skin disease but to date, it remains unclear how these cytokines are released. Though *extracellular* stimulation of keratinocytes with high concentrations of the double-stranded RNA (dsRNA) synthetic analogue Poly(I:C) was shown to induce IL-36γ release from keratinocytes [23], genome-wide association studies have shown a link between the genes encoding the dsRNA *intracellular* sensors retinoic acid-inducible gene I (RIG-I) and melanoma differentiation-associated protein 5 (MDA5) with an increased risk of psoriasis vulgaris [24–26]. NLRP1 is the main inflammasome sensor in human keratinocytes [27] detecting intracellular dsRNA [28] and polymorphisms in this gene have been associated with psoriasis [29]. It was therefore of interest to verify whether the presence of dsRNA in the cytoplasm could also induce IL-36 cytokine release by activating intracellular sensors.

In this study, we demonstrate that transfection of dsRNA is a potent inducer of IL-36γ release from human keratinocytes. Intracellular dsRNA stimulates cleavage of apoptotic and inflammatory caspases resulting in keratinocyte cell death, the upregulation of Interferon-stimulated genes (ISGs) and inflammasome activation. IL-36γ released from primary human keratinocytes in response to transfected dsRNA is associated with cell death and is processed to its mature form extracellularly, in the absence of neutrophil proteases.

The cationic aliphatic antimicrobial peptide, LL37, a key molecule in triggering early events in the pathogenesis of psoriasis vulgaris, can facilitate cell entry by binding to extracellular Poly(I:C) and activate the TLR3 and MAVS pathways in keratinocytes [30, 31]. Here we show that LL37/dsRNA complexes licence IL-36γ release in the absence of significant cell death by activating RIG-I-like receptor signalling that induces the release of full-length IL-36γ, involving cleavage of Caspase-3 and GSDME pore formation.

## Results

### Intracellular Poly(I:C) potently induces IL-36γ release from keratinocytes, which is subsequently cleaved extracellularly

**Fig. 1:**
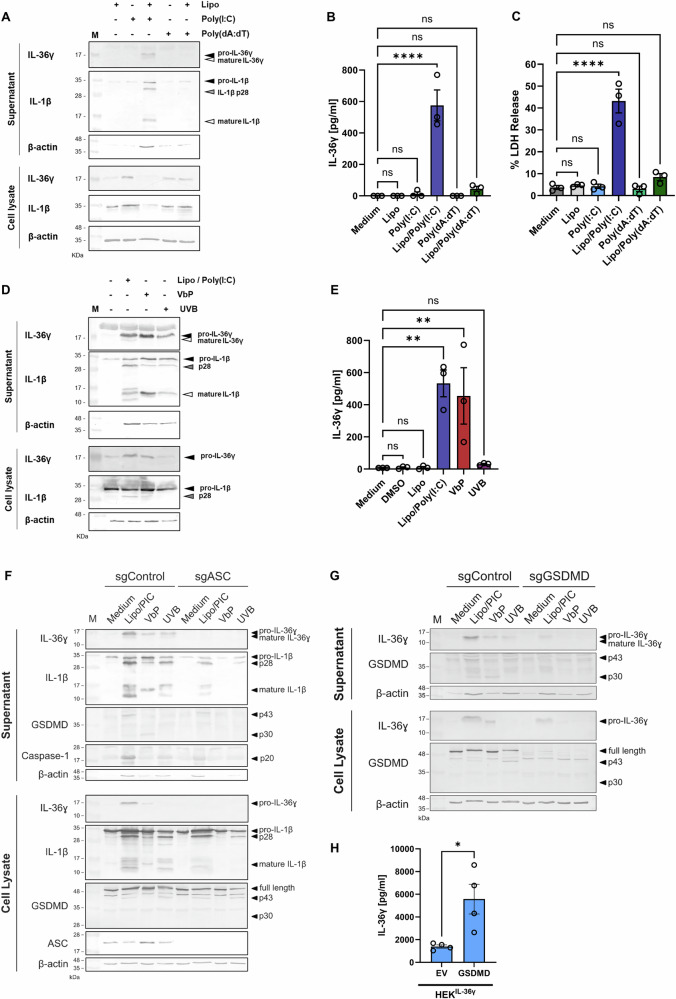
Intracellular dsRNA analogue Poly(I:C) is a potent inducer of IL-36γ release from human primary keratinocytes. **A**–**C** Human primary keratinocytes (HPKs) were stimulated or transfected with Poly(I:C) or Poly(dA:dT) (1 μg/ml) for 24 h. **A** Cell lysates and supernatants were subjected to SDS-PAGE and immunoblotting with indicated antibodies. Supernatants were collected and assayed for **B** IL-36γ release by ELISA or subjected to **C** lactate dehydrogenase (LDH) assay analysis. **D**, **E** HPKs were transfected with Poly(I:C) (1 μg/ml), stimulated with Val-boroPro (VbP, 1 μM), one UVB pulse (0.0875 J/cm^2^) or DMSO and incubated for 24 h. **D** Cell lysates and supernatants were subjected to SDS-PAGE and immunoblotting with indicated antibodies. **E** Supernatants were collected and assayed for IL-36γ release by ELISA. **F**, **G** *ASC- or GSDMD-*deficient N/TERT-1 cell-lines (sgASC / sgGSDMD) were stimulated with LL37/Poly(I:C) (5:5 μg/ml) complexes, VbP (0.5 μM), transfected Poly(I:C) (PIC, 1 μg/ml), or one UVB pulse (0.0875 J/cm^2^) for 18 h and cell lysates and supernatants were subjected to SDS-PAGE and immunoblotting with indicated antibodies. **H** HEK293^IL-36γ^ cells were transfected with GSDMD or empty vector (EV) (1 μg) for 24 h. IL-36γ levels in supernatants were measured by ELISA. Data are presented as (**A**, **D**, **F**, **G**) representative of three independent experiments, (**B**, **C**, **E**, **H**) presented as the mean ±S.E.M. of 3–4 independent experiments and analysed with (**B**, **C**, **E**) one-way ANOVA followed by Dunnett’s multiple comparisons test or (**H**) a two-tailed t test. *p < 0.05, **p < 0.01, ***p < 0.001, ****p < 0.0001. ns non-significant, Lipo Lipofectamine 2000, M protein marker, PIC Poly(I:C), sg single-guide RNA.

Transfection of human primary keratinocytes (HPKs) with Poly(I:C) using liposomal transfection reagent induces both IL-36γ expression and subsequent release (Fig. 1A, B and Supplementary Fig. 1A). This is associated with a significant increase in cell death, as demonstrated by LDH release (Fig. 1C). Notably, a faster migrating IL-36γ band, corresponding to mature IL-36γ, is detectable in supernatants but not cell lysates of Poly(I:C)-transfected HPKs, indicating that IL-36γ release is accompanied by extracellular proteolytic processing by keratinocyte-derived proteases (Fig. 1A and Supplementary Fig. 1B). At the same concentration, non-transfected Poly(I:C) treatment fails to promote IL-36γ expression and release at 24 h (Fig. 1A, B).

Direct and indirect activation of the NLRP1 inflammasome by Poly(I:C) and the DNA analogue Poly(dA:dT), respectively induces IL-1β release in unprimed keratinocytes [28, 32], however Poly(dA:dT) induced only weak IL-1β release at the time-points tested here (Fig. 1A and Supplementary Fig. 1C). Intracellular Poly(I:C) was a more potent inducer of IL-36γ expression and release than Poly(dA:dT) (Fig. 1A, B). Other NLRP1 agonists were tested to determine if inflammasome assembly facilitates IL-36 cytokine release. Primary keratinocytes were subjected to stimulation with the DPP8/9 inhibitor Val-boroPro (VbP) or UVB and were incubated for 24 h. Similar to intracellular Poly(I:C), VbP induced potent IL-36γ protein expression and release, whereas UVB induced only mild IL-36γ release (Fig. 1D, E). VbP induced more IL-1β release than Lipo/Poly(I:C) transfection but less cell death as measured by LDH assay (Supplementary Fig. 1D, E). In line with this, all three NLRP1 agonists demonstrate differential cleavage of apoptotic caspases (Supplementary Fig. 1F). Notably, multiple cleaved forms of IL-1β are observed (Fig. 1A), including the 28 kDa fragment, which can be generated by Caspase-1, Caspase-4 or Caspase-7 cleavage at Asp26 [33–35].

Release of IL-36γ in response to NLRP1 agonists is lost in ASC-deficient N/TERT-1 cells (Fig. 1F). IL-36γ release is also reduced in GSDMD-deficient N/TERT-1 cells in response to transfected Poly(I:C), though not completely abrogated (Fig. 1G), whereas VbP- and UVB-induced IL-36γ release were undetectable in the absence of GSDMD. GSDMD is processed to the pore-forming p30 fragment upon VbP treatment, whereas the inactive p43 fragment is predominant in N/TERT-1 control cells transfected with Poly(I:C) or stimulated with UVB. HEK293 cells were generated to constitutively express full-length IL-36γ (HEK^IL-36γ^). Overexpression of GSDMD in these cells results in its spontaneous cleavage and the subsequent release of IL-36γ (Fig. 1H, Supplementary Fig. 1G). This demonstrates that full-length IL-36γ can exit GSDMD pores.

NLRP1 inflammasome activation facilitates the release of IL-36γ from HPKs, however, as IL-36γ lacks a Caspase-1 cleavage site, it is not cleaved intracellularly. Extracellular proteolytic processing of IL-36γ correlates with lytic cell death (Supplementary Fig. 1H). Intracellular dsRNA induces a form of inflammatory cell death in keratinocytes that releases IL-36γ and allows processing of the cytokine extracellularly to release its full biological activity in the absence or prior to neutrophil infiltration.

### Antimicrobial peptide LL37 complexed with dsRNA triggers IL-36γ release from human primary keratinocytes

**Fig. 2:**
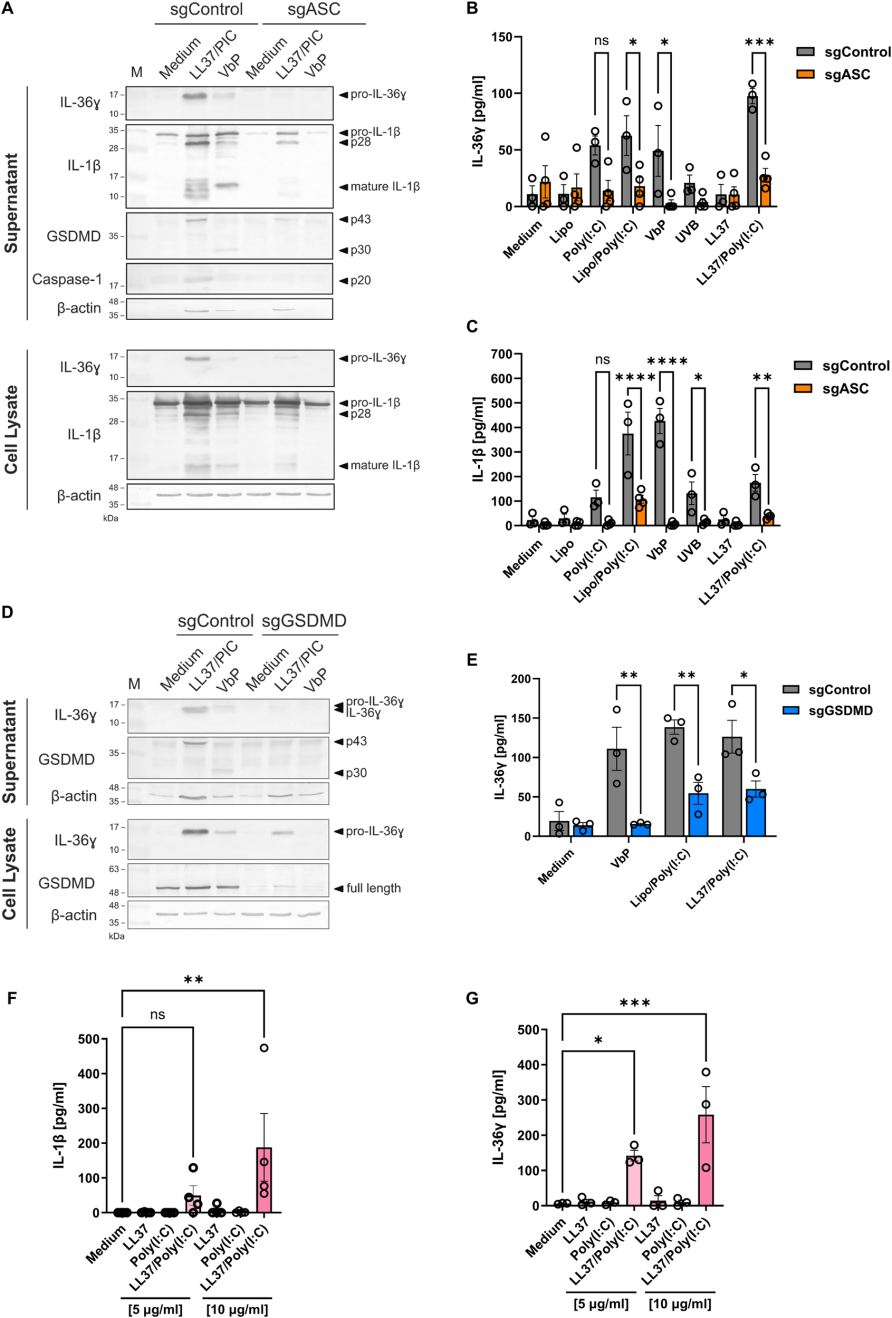
LL37/Poly(I:C) complexes trigger inflammasome activation and IL-36γ release. **A**–**C** *ASC-*deficient N/TERT-1 cells (sgASC) were stimulated with LL37/Poly(I:C) (5:5 μg/ml) complexes or separately (each 5 μg/ml), VbP (0.5 μM), transfected Poly(I:C) (1 μg/ml), or one UVB pulse (0.0875 J/cm^2^) for 18 h and **A** cell lysates and supernatants were subjected to SDS-PAGE and immunoblotting with indicated antibodies or supernatants were analysed by ELISA for **B** IL-36γ and **C** IL-1β levels. **D**, **E** *GSDMD-*deficient N/TERT-1 cells (sgGSDMD) were stimulated with LL37/Poly(I:C) (5:5 μg/ml) complexes or VbP (1 μM) for 18 h and **D** cell lysates and supernatants were subjected to SDS-PAGE and immunoblotting with indicated antibodies or **E** supernatants were analysed by ELISA for IL-36γ levels. **F**, **G** HPKs were stimulated with LL37/Poly(I:C) (5:5 μg/ml or 10:10 μg/ml) complexes for 24 h. Supernatants were analysed for the release of **F** IL-1β and **G** IL-36γ by ELISA. Data are presented as a representative (**A**, **D**) of three independent experiments or are presented (**B**, **C**, **E**–**G**) as the mean ±S.E.M. of at least 3–4 independent experiments and subjected to (**B**, **C**, **E**) two-way ANOVA followed by Šidák’s multiple comparisons test or (**F**, **G**) one-way ANOVA followed by Dunnett’s multiple comparisons test. *p < 0.05, **p < 0.01, ***p < 0.001, ****p < 0.0001. ns non-significant, Lipo Lipofectamine 2000, M protein marker, PIC Poly(I:C), sg single-guide RNA.

Previously, it was reported that the cationic antimicrobial cathelicidin peptide LL37 facilitates entry of Poly(I:C) into primary keratinocytes and induces a type I Interferon response [30]. To determine whether LL37/Poly(I:C) complexes could induce IL-36γ release, Poly(I:C) was incubated together with synthetic LL37 for 15 min prior to addition to HPKs. LL37 facilitates entry of Poly(I:C) into primary keratinocytes and activates a type IFN I response (Supplementary Fig. 2A, B), as previously described [30, 36]. LL37/Poly(I:C) complexes also increase IL-36γ transcript levels in HPKs (Supplementary Fig. 2C). In N/TERT-1 cells, LL37/Poly(I:C) treatment results in the release of IL-36γ, which is diminished in the absence of ASC (Fig. 2A, B). LL37/Poly(I:C) complexes also weakly induce IL-1β release in N/TERT-1 cells in an ASC-dependent manner (Fig. 2A, C). LL37/Poly(I:C) triggers only limited LDH release, which is lower in ASC-deficient cells, albeit not significantly (Supplementary Fig. 2D). Additionally, IL-36γ release in response to LL37/Poly(I:C) stimulation is reduced in GSDMD-deficient N/TERT-1 cells (Fig. 2D, E). Since LDH and IL-1β release were weak in response to LL37/Poly(I:C) complexes, no significant reduction was detected in GSDMD-deficient N/TERT-1 cells (Supplementary Fig. 2E–G), which coincides with the absence of a significant difference in PI positivity and weak IL-1β release (Supplementary Fig. 2F, G).

In HPKs, LL37/Poly(I:C) complexes only release minimal IL-1β or LDH (Fig. 2F, Supplementary Fig. 2H). We speculated that this was a threshold response and a modest increase in the concentration of LL37/Poly(I:C) complexes (to 10 μg/ml) induces significant IL-1β and LDH release. At both concentrations, however, LL37/Poly(I:C) complexes induce IL-36γ release (Fig. 2G). Treatment with LL37 or Poly(I:C) alone failed to induce IL-36γ release in HPKs. Interestingly, only full-length IL-36γ and not the mature form, was detectable in supernatants from LL37/Poly(I:C)-stimulated HPKs (Supplementary Fig. 2I). Keratinocyte-derived cathepsin S, a protease of full-length IL-36γ [10] is only present in supernatants in response to liposomal-transfected Poly(I:C), but not LL37/Poly(I:C) complexes (Supplementary Fig. 2J), consistent with the lack of significant LDH release at these concentrations in HPKs (Supplementary Fig. 2H).

These data demonstrate that cathelicidin peptide LL37 complexed to dsRNA only triggers modest inflammasome activation in keratinocytes as part of a threshold response, while the N/TERT-1 cell-line is more sensitive to stimulation. In the absence of inflammasome activation, IL-36γ release remains unaffected suggesting that another pathway is involved in its liberation in response to LL37/Poly(I:C) complexes.

### LL37/Poly(I:C) complexes activate Caspase-3 and GSDME pore formation in human primary keratinocytes

**Fig. 3:**
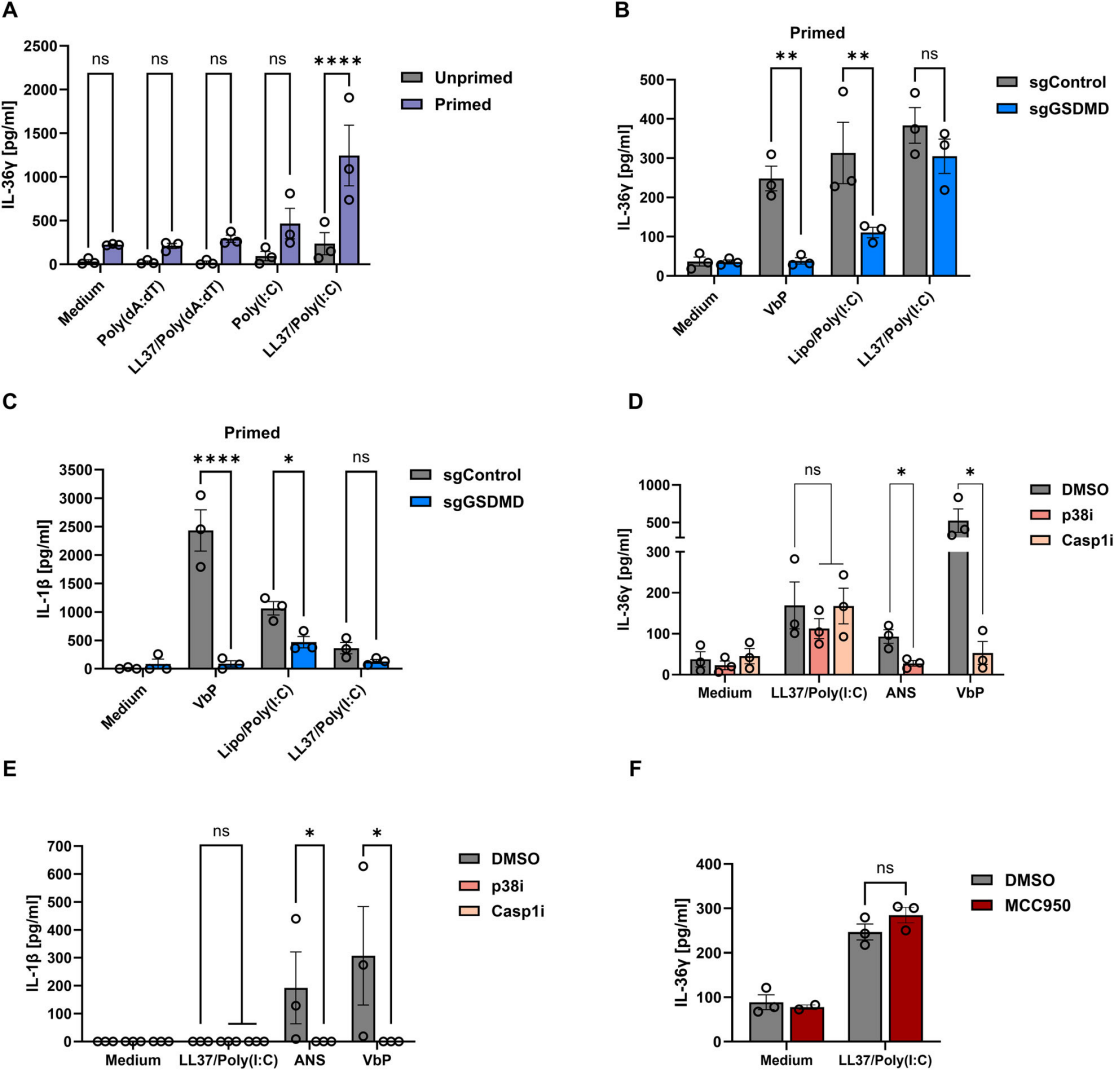
LL37/Poly(I:C) complexes induce IL-36γ release from keratinocytes independent of inflammasome and GSDMD activity. **A** HPKs were primed with IL-17A (100 ng/ml) or left unprimed (medium control) for 6 h prior to stimulation with Poly(I:C), Poly(dA:dT), LL37/Poly(I:C) or LL37/Poly(dA:dT) complexes (all 5 μg/ml) for 18 h. Supernatants were analysed for IL-36γ release by ELISA. **B**, **C** *GSDMD-*deficient N/TERT-1 cells (sgGSDMD) were stimulated with IL-17A (100 ng/ml) for 6 h prior to stimulation with LL37/Poly(I:C) complexes (5 μg/ml), VbP (1 μM), or transfected with Poly(I:C) (1 μg/ml) for 18 h. Supernatants were analysed for **D** IL-36γ or **E** IL-1β levels by ELISA. **D**, **E** HPKs were stimulated with IL-17A (10 ng/ml) and TNF-α (5 ng/ml) for 5 h, followed by inhibitor treatment with SB 203580 (p38i, 20 μM), Belnacasan (Casp1i, 10 μM), or DMSO for 1 h before stimulation with LL37/Poly(I:C) complexes (5 μg/ml), VbP (1 μM), or anisomycin (ANS, 1 μM) for 18 h. Supernatants were measured for **D** IL-36γ or **E** IL-1β by ELISA. **F** HPKs were primed with IL-17A for 5 h, followed by inhibitor treatment with MCC950 or DMSO for 1 h before stimulation with LL37/Poly(I:C) complexes for 18 h. Cells were treated with MCC950 1 h before stimulation. Supernatants were measured for IL-36γ release by ELISA. Data are presented (**A**–**F**) as the mean ±S.E.M. of 3 independent experiments and analysed with (**A**–**C**, **F**) two-way ANOVA and Šidák’s or (**D**, **E**) two-way ANOVA followed by Dunnett’s multiple comparisons test. *p < 0.05, **p < 0.01, ****p < 0.0001. ns non-significant, Lipo Lipofectamine 2000, M protein marker, sg single-guide RNA.

Notably, in ASC- and GSDMD-deficient N/TERT-1 cells, IL-36γ expression is also reduced (Fig. 2A, D). This could indicate that inflammasome activation promotes IL-36γ expression in a positive feedback loop, due to the release of IL-1β or danger-associated molecular patterns (DAMPs). To determine whether inflammasome activation and GSDMD pore formation are indeed required for the release of IL-36γ, cells were primed to increase expression of IL-36γ prior to stimulation with LL37/Poly(I:C) complexes. Treatment with the psoriasis-associated cytokine, IL-17A, enhances IL-36γ protein expression (Supplementary Fig. 3A). Priming IL-36γ expression for 6 h with IL-17A prior to treatment with LL37/Poly(I:C) significantly increased the amount of IL-36γ released from HPKs (Fig. 3A), while IL-17A treatment alone does not induce IL-36γ or LDH release (Fig. 3A, Supplementary Fig. 3B).

In primed cells, LL37 complexed to the DNA analogue Poly(dA:dT), failed to induce a potent IL-36γ response (Fig. 3A), suggesting that RNA sensor activation is crucial for IL-36γ release. Additionally, the release of IL-36α was undetectable in response to LL37/Poly(I:C) and LL37/Poly(dA:dT) complexes (Supplementary Fig. 3C).

GSDMD-deficient N/TERT-1 cells were pretreated with IL-17A for 6 h prior to stimulation with LL37/Poly(I:C) complexes (Fig. 3B, C). IL-36γ release remains intact in GSDMD-deficient cells stimulated with LL37/Poly(I:C) complexes in primed cells. IL-36γ and IL-1β release from cells transfected with Poly(I:C) or treated with VbP are still significantly reduced in GSDMD-deficient cells, despite priming of IL-36γ expression (Fig. 3B, C) and LDH release is significantly decreased in response to VbP (Supplementary Fig. 3D).

Since GSDMD is dispensable for IL-36γ release in response to LL37/Poly(I:C) complexes, it was of interest to assess whether NLRP1 activation is required. NLRP1 can be activated by phosphorylation via p38 MAPK, downstream of ZAKα and other kinases [37, 38]. LL37/Poly(I:C) complexes induce weak phosphorylation of p38 MAPK (Supplementary Fig. 3E). Inhibition of p38 MAPK and Caspase-1, respectively, abrogates Anisomycin- (ANS) and VbP-induced IL-36γ, IL-1β and LDH release (Fig. 3D, E, Supplementary Fig. 3F). However, p38 MAPK and Caspase-1 inhibition have no significant effect on LL37/Poly(I:C)-induced IL-36γ release in keratinocytes primed with IL-17A (Fig. 3D). Additionally, treatment with the NLRP3 inhibitor, MCC950, fails to inhibit LL37/Poly(I:C)-induced IL-36γ and LDH release in primed HPKs (Fig. 3F, Supplementary Fig. 3G), suggesting inflammasome activation is not involved in this response.

**Fig. 4:**
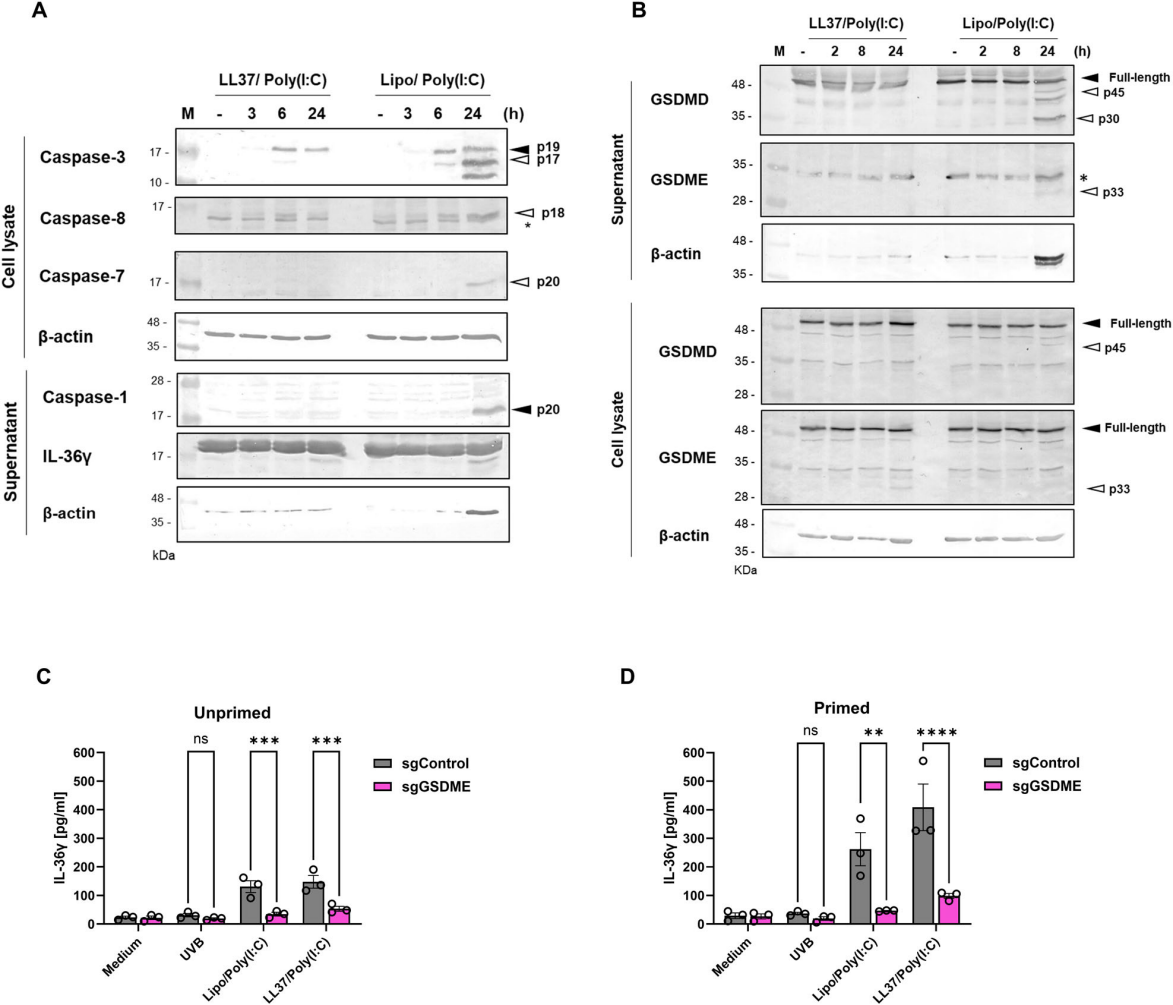
LL37/Poly(I:C) complexes activate Caspase-3 and GSDME cleavage in human primary keratinocytes. **A**, **B** HPKs were stimulated with LL37/Poly(I:C) complexes (5 μg/ml) or were transfected with Poly(I:C) (1 μg/ml) for indicated times control treatment (−) indicates Medium or Lipo, respectively. Supernatants and cell lysates were subjected to SDS-PAGE and immunoblotting with indicated antibodies. * indicates non-specific band (**C**, **D**) *GSDME-*deficient N/TERT-1 cells (sgGSDME) were (**D**) primed with IL-17A (100 ng/ml) or (**C**) left unprimed for 6 h prior to transfection with Lipo/Poly(I:C) (1 μg/ml), stimulation with LL37/Poly(I:C) complexes (5 μg/ml) or one UVB pulse (0.0875 J/cm^2^) for 18 h. Data are presented as a representative (**A**, **B**) of three independent experiments or are presented (**C**, **D**) as the mean ±S.E.M. of 3 independent experiments and analysed with two-way ANOVA and Šidák’s multiple comparisons test. **p < 0.01, ***p < 0.001, ****p < 0.0001. ns non-significant, Lipo Lipofectamine 2000, M protein marker, sg single-guide RNA.

To evaluate caspase activation, HPKs were stimulated with LL37/Poly(I:C) complexes for 3, 6 and 24 h and cleavage of caspases was assessed (Fig. 4A). Caspase-3 cleavage, as identified by the p19 and p17 cleavage-fragments, was present in Lipo/Poly(I:C)- and LL37/Poly(I:C)-stimulated keratinocytes, though only exposure to the former triggered sustained Caspase-3 auto-catalysis to the pro-apoptotic p17 fragment (Fig. 4A). Caspase-3 p17 was observable in keratinocytes stimulated with LL37/Poly(I:C) at 6 h but was not evident at later time points. Moreover, cleavage of Caspase-8 decreases at 24 h in response to LL37/Poly(I:C) stimulation, while Caspase-8 cleavage was more evident at 24 h in Lipo/Poly(I:C)-transfected keratinocytes. Caspase-1 was only found in the supernatants of keratinocytes transfected with Poly(I:C), consistent with inflammasome activation and IL-1β release in response to this treatment in HPKs.

Caspase-3 is capable of cleaving GSDME into its pore-forming fragment and therefore it was of interest to assess whether GSDME plays a role in IL-36γ release in response to LL37/Poly(I:C) complexes. In HPKs, liposomal-transfected Poly(I:C) induces GSDMD cleavage to both the inactive p43 and pore-forming p30 fragments as evident in supernatants (Fig. 4B), whereas only GSDMD p43 cleavage is observable in cell lysates of HPKs stimulated with LL37/Poly(I:C) complexes. GSDME cleavage is present in cell lysates in response to both stimuli, concordant with Caspase-3 cleavage, however, the N-terminal GSDME fragment was not detectable in the supernatants of LL37/Poly(I:C)-treated HPKs, consistent with the lack of cell death in response to this ligand.

In GSDME-deficient N/TERT-1 cells (Supplementary Fig. 4A) the release of IL-36γ is significantly reduced in response to LL37/Poly(I:C) in both unprimed and primed cells, indicating that GSDME plays an important role in expediting IL-36γ release in response to LL37/Poly(I:C) complexes (Fig. 4C, D). Similarly, IL-36γ and LDH release is significantly decreased in response to liposomal transfected Poly(I:C) in unprimed and primed cells (Fig. 4C, D, Supplementary Fig. 4B). Collectively, these data demonstrate that LL37/Poly(I:C) complexes initiate Caspase-3 and GSDME cleavage to release IL-36γ, independent of inflammasome activation.

### Priming of keratinocytes with psoriasis-associated cytokines induce RIG-I-like receptor protein expression and expedite IL-36γ release in response to LL37/Poly(I:C) complexes

**Fig. 5:**
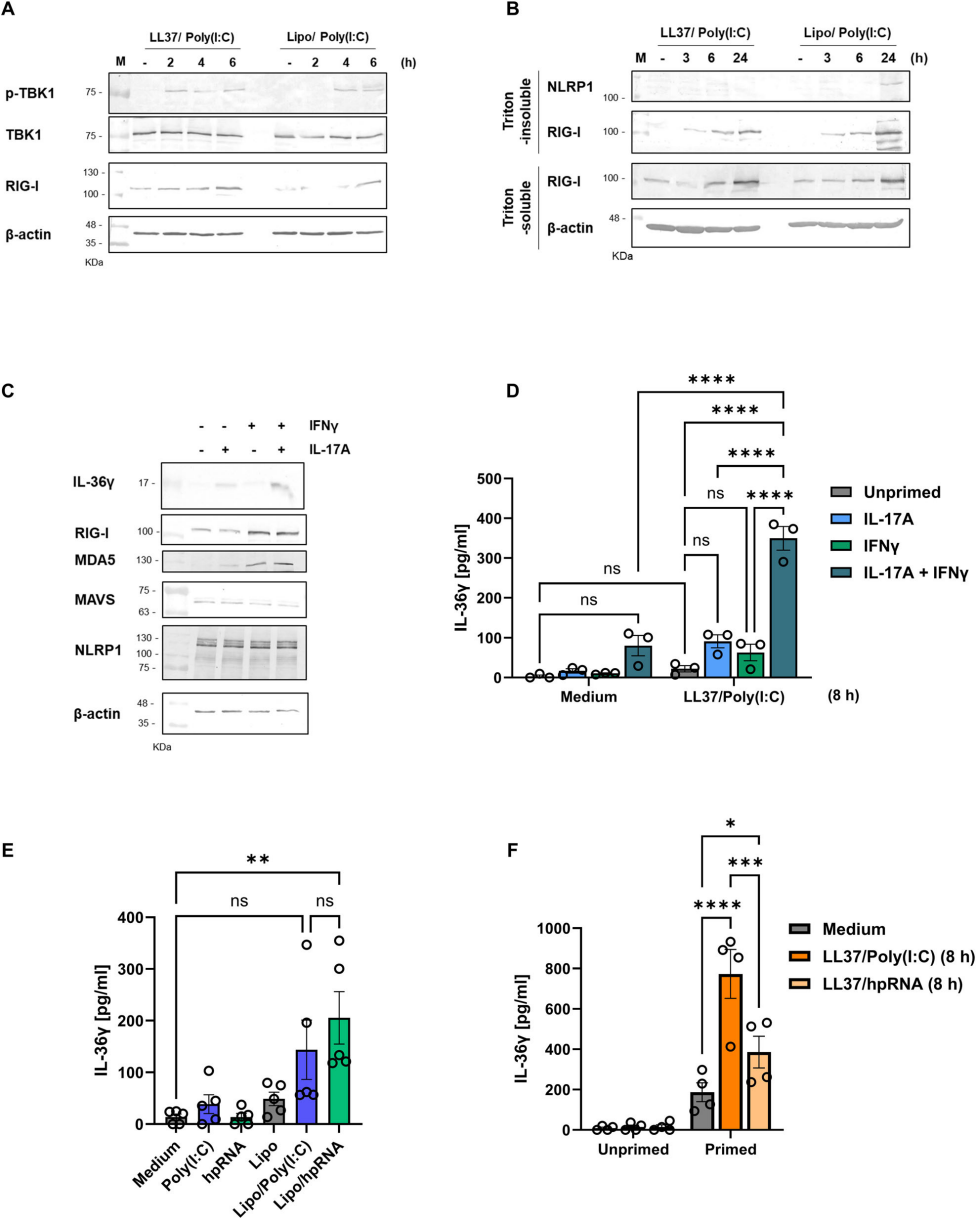
Priming of keratinocytes induces RLR expression and rapid release of IL-36γ in response to LL37/Poly(I:C) complexes. **A**, **B** HPKs were stimulated with LL37/Poly(I:C) complexes (5 μg/ml) or were transfected with Poly(I:C) (1 μg/ml) for indicated times (control treatment (−) indicates Medium or Lipo, respectively). **A** Cell lysates or **B** Triton-soluble fractions and -insoluble fractions were subjected to SDS-PAGE and immunoblotting with indicated antibodies. **C**, **D** HPKs were primed with IL-17A (100 ng/ml) and/or IFNγ (10 ng/ml) for 16 h, followed by (**D**) stimulation with LL37/Poly(I:C) complexes for 8 h. **C** Cell lysates assay were subjected to SDS-PAGE and immunoblotting with indicated antibodies. **D** Supernatants were measured for IL-36γ release by ELISA. **E** HPKs were primed with IL-17A (100 ng/ml) for 6 h prior to liposomal transfection of Poly(I:C) (1 μg/ml) or hpRNA (1 μg/ml) for 18 h. Supernatants were analysed for IL-36γ release by ELISA. **F** HPKs were primed with IL-17A (100 ng/ml) and IFNγ (10 ng/ml) for 16 h, followed by stimulation with LL37/Poly(I:C) or LL37/hpRNA complexes (both 5 μg/ml) for 8 h. Supernatants were subjected to ELISA to measure IL-36γ levels. Data are presented as a representative (**A**–**C**) of three independent experiments or are presented (**D**–**F**) as the mean ±S.E.M. of at least 3 independent experiments and are subjected to two-way ANOVA followed by (**D**) Tukey’s or (**E, F**) Šidák’s multiple comparisons test. *p < 0.05, **p < 0.01, ***p < 0.001, ****p < 0.0001. ns non-significant, Lipo Lipofectamine 2000, M protein marker.

Since LL37/Poly(I:C) complexes induce a type I interferon response (as shown in Supplementary Fig. 2B) and phosphorylation of TBK1 (Fig. 5A), we hypothesised that the RIG-I-like receptor (RLR) signalling pathway might be responsible for inducing IL-36γ release in response to these complexes. Indeed, RIG-I was detectable in the insoluble fractions of keratinocytes stimulated with LL37/Poly(I:C) or transfected with Poly(I:C) (Fig. 5B), suggesting it engages in complex formation.

As expression of RLRs and IL-36γ are low in HPKs but increased upon LL37/Poly(I:C) stimulation (Fig. 5A, B, Supplementary Fig. 2C), priming with IFNγ could be used to induce RLR expression allowing a more potent and rapid response to LL37/Poly(I:C) complexes. To test this, HPKs were co-primed for 16 h with IL-17A and IFNγ to induce IL-36γ and RLR expression, respectively (Fig. 5C). Neither cytokine had an effect on the expression levels of NLRP1 nor the RLR adaptor, MAVS. Subsequently, HPKs were stimulated with LL37/Poly(I:C) complexes for 8 h (Fig. 5D). In response to LL37/Poly(I:C) complexes in unprimed cells, there is no significant IL-36γ release at 8 h. However, in cells co-primed with IL-17A and IFNγ, LL37/Poly(I:C) complexes trigger significant release of IL-36γ. Priming with IL-17A and IFNγ separately had no consequential effect on LL37/Poly(I:C)-induced IL-36γ release. These data suggest that upregulation of RLRs might licence IL-36γ release in response to LL37/Poly(I:C) complexes.

To determine whether specific activation of RLRs could induce IL-36γ release as well as a type I interferon response, HPKs were primed with IL-17A for 6 h and subsequently transfected with a specific RIG-I agonist, 5’-triphosphate hairpin RNA (hpRNA) to observe whether activation of RIG-I could lead to IL-36γ release (Fig. 5E). Transfected hpRNA is capable of inducing significant IL-36γ release, indicating that RIG-I activation can expedite the release of IL-36γ. LDH release though increased was not significant, yet transfected cells showed increased PI positivity (Supplementary Fig. 5A, B). More CXCL10 release was induced in response to transfected hpRNA than Poly(I:C) (Supplementary Fig. 5C).

Furthermore, priming HPKs with IFNγ and IL-17A and stimulating with LL37/hpRNA complexes triggered the early release of IL-36γ after 8 h (Fig. 5F), while CXCL10 production doesn’t require priming (Supplementary Fig. 5D). LDH release from HPKs is significantly upregulated, albeit still at low levels in response to LL37/Poly(I:C) (Supplementary Fig. 5E).

### RLR and MAVS activation by LL37/Poly(I:C) complexes induces GSDME cleavage and IL-36γ release

**Fig. 6:**
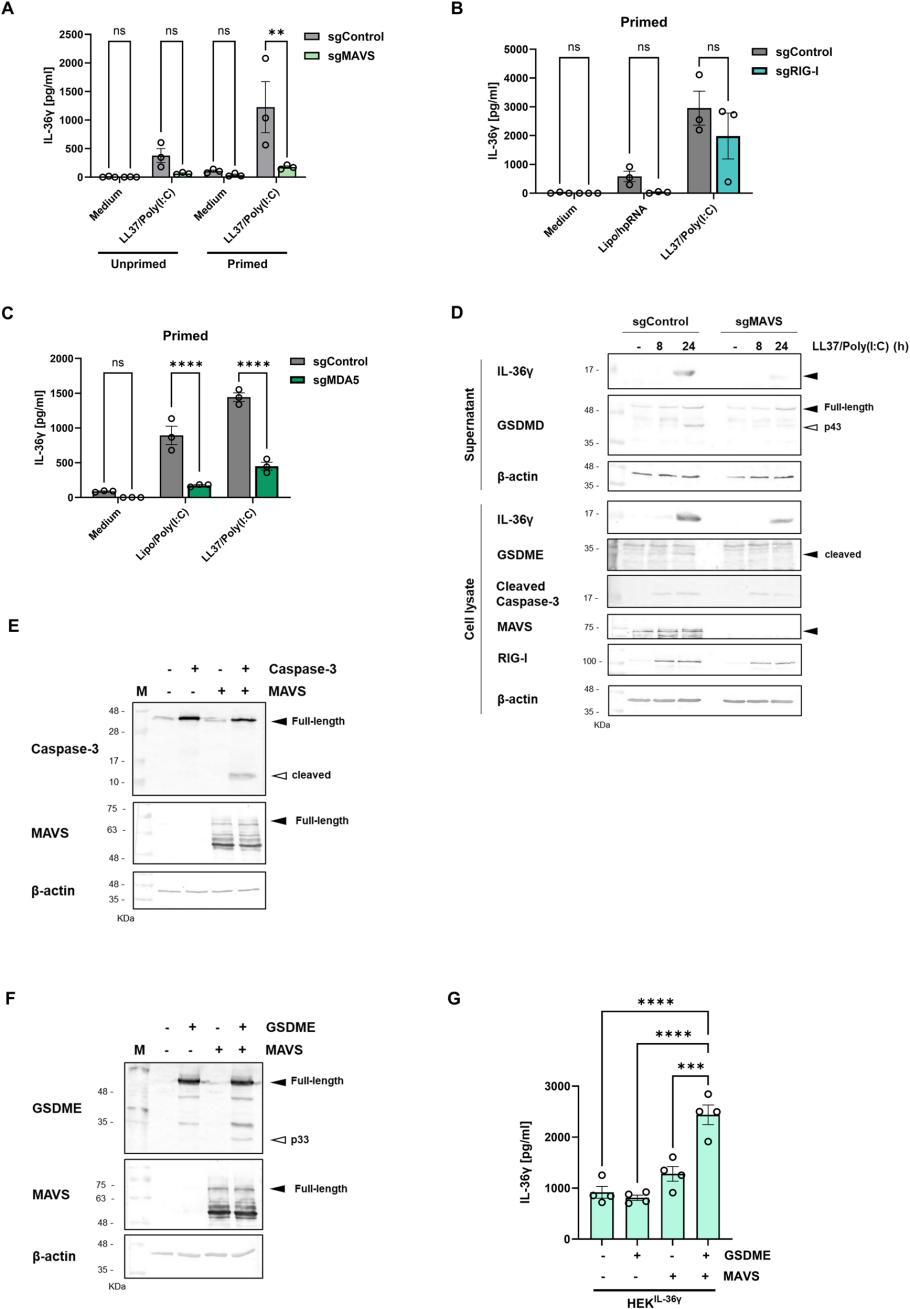
RLR/MAVS pathway activation induces Caspase-3 cleavage and GSDME-mediated IL-36γ release. **A** *MAVS-*, **B** *RIG-I-* or **C** *MDA5-*deficient N/TERT-1 cell-lines (sgMAVS/sgRIG-I/sgMDA5) were primed with IL-17A (100 ng/ml) or medium (unprimed) for 6 h prior to treatment with LL37/Poly(I:C) complexes (5 μg/ml) or transfection with either Poly(I:C) or hpRNA (both 1 μg/ml) for 18 h. Supernatants were analysed for IL-36γ release by ELISA. **D** *MAVS-*deficient N/TERT-1 cells (sgMAVS) were stimulated with LL37/Poly(I:C) complexes (5 μg/ml) at indicated times. Control treatment (-) indicates Medium only. Cell lysates were subjected to SDS-PAGE and immunoblotting with indicated antibodies. HEK293 cells were transfected with plasmids encoding empty vector (−), **E** Caspase-3 or **F** GSDME with and without MAVS for 24 h. Cell lysates were subjected to SDS-PAGE and immunoblotting with indicated antibodies. **G** HEK293^IL-36γ^ cells were transfected with empty vector (−) or GSDME with and without MAVS for 24 h and supernatants were measured for IL-36γ release by ELISA. Data are presented as a representative (**D**–**F**) of 3 independent experiments or are presented as mean ±S.E.M. of at least 3 independent experiments and subjected to a **A**–**C** two-way ANOVA followed by Šidák’s or **G** one-way ANOVA followed by Tukey’s multiple comparisons test. **p < 0.01, ***p < 0.001, ****p < 0.0001. ns non-significant. Lipo Lipofectamine 2000, sg single-guide RNA.

As full-length MAVS forms prion-like aggregates upon RLR activation to mediate downstream signalling [39], we sought to investigate whether LL37/Poly(I:C)-induced IL-36γ expression and release would be inhibited in N/TERT-1 cells lacking full-length MAVS (Supplementary Fig. 6A). In IL-17A-primed MAVS-deficient cells, release of IL-36γ and LDH is reduced in response to LL37/Poly(I:C) stimulation (Fig. 6A, Supplementary Fig. 6B).

To assess LL37/Poly(I:C) binding to RLRs, HPKs were stimulated with Biotinylated-Poly(I:C) complexed to LL37 or transfected using liposomal reagent. Biotinylated-Poly(I:C) transfected or complexed to LL37 is capable of binding directly to RIG-I indicating that this RLR is activated upon treatment (Supplementary Fig. 6C). IL-36γ release is lost in response to transfected RIG-I agonist (albeit not statistically significant), hpRNA in RIG-I-deficient N/TERT-1 cells, however, IL-36γ release upon LL37/Poly(I:C) complex stimulation was not significantly affected (Fig. 6B) and LDH release was also not reduced (Supplementary Fig. 6D**)**. This may be due to redundancy involving MDA5 binding to LL37/Poly(I:C) complexes. In MDA5-deficient cells (Supplementary Fig. 6E**)**, IL-36γ release was significantly reduced in response to LL37/Poly(I:C) complexes (Fig. 6C), however, this response was not completely lost, which might be due to the compensatory action of RIG-I, which does get activated by LL37/Poly(I:C) complexes (Fig. 5B, Supplementary Fig. 6C**)**. Similar to RIG-I-deficient cells, LDH release is also not reduced in cells lacking MDA5 (Supplementary Fig. 6E).

Since the RLR-MAVS pathway is essential for LL37/Poly(I:C)-induced IL-36γ release, we sought to assess the role of MAVS in GSDME activation in response to LL37/Poly(I:C) complexes. Unprimed MAVS-deficient cells were stimulated with LL37/Poly(I:C) complexes for 8 and 24 h (Fig. 6D). In addition to release, LL37/Poly(I:C)-induced expression of IL-36γ and RIG-I are also reduced in non-primed MAVS-deficient cells. Caspase-3 is predominantly processed to the p19 fragment as in HPKs (as shown in Fig. 4A) and cleavage is reduced in MAVS-deficient cells at 24 h upon stimulation. Moreover, there is reduced cleavage of Caspase-3 substrates GSDME and GSDMD p43 in lysates and supernatants, respectively.

Overexpression of MAVS in HEK293 and HEK^IL-36γ^ cells induces Caspase-3 cleavage (Fig. 6E) and the generation of the GSDME N-terminal fragment (Fig. 6F, Supplementary Fig. 6F). Generation of the GSDME pore-forming fragment, facilitates IL-36γ release (Fig. 6G) and induces an increase in cell death (Fig. 6G, Supplementary Fig. 6G).

These data indicate that LL37/dsRNA complexes induce IL-36γ release dependent on the RLR/MAVS signalling axis, which triggers Caspase-3 activation and GSDME pore formation.

## Discussion

Previously, it has been described that extracellular stimulation of keratinocytes with dsRNA analogue Poly(I:C) induces Caspase-3-mediated cell death and release of IL-36γ [23]. Unlike other members of the IL-1 family, IL-36 cytokines lack Caspase-1 cleavage motifs so their processing is independent of inflammasome activation [5]. In our hands, at lower concentrations than previously used, extracellular stimulation with Poly(I:C) fails to induce IL-36γ release, however, liposomal delivery of Poly(I:C) induces a potent response, that is partially mediated by inflammasome activation. Additionally, overexpression of GSDMD and its spontaneous cleavage facilitates IL-36γ release in HEK293 cells. Notably, ectopic expression of wild-type GSDMD previously did not induce pyroptosis in HEK293, which might be due to a difference in concentrations used or the N-terminal location of the epitope tag that potentially hinders pore formation [40]. IL-36 cytokines require no change in charge for membrane localisation like IL-1β cytokines [41, 42] and cleavage of GSDMD was sufficient to release full-length IL-36γ. IL-36 cytokines are primarily cleaved extracellularly and this function is performed by neutrophil proteases, however, keratinocyte cell disruption associated with liposomal transfection of Poly(I:C) releases proteases that are capable of cleaving IL-36γ extracellularly prior to the recruitment of neutrophils.

The expression of IL-36 cytokines at barrier sites highlights the importance of this cytokine family at mounting an immune response to infection. Extracellular cleavage of IL-36 cytokines can be advantageous over intracellular processed IL-1β and IL-18 in response to viral infection. Virally-infected cells that undergo cell lysis might release IL-36 cytokines that can then be activated extracellularly. It is hypothesised that IL-36 cytokines might have arisen by gene duplication due to evolutionary pressure influenced by viral immuno-evasive strategies that inhibit or circumvent inflammasome and IL-1β activity [43]. Indeed, GPP patients show varying degrees of a type I Interferon transcriptomic signature that is potentiated by IL-36γ [44].

In GPP, a role for LL37 has not yet been clearly described but is thought to play a role due to its upregulation of chemokines, CXCL1 and IL-8 associated with neutrophil infiltration [45, 46]. In psoriasis vulgaris, LL37 and other cationic antimicrobial peptides bind to self-nucleic acids from damaged keratinocytes and activate plasmacytoid dendritic cells, thus initiating early steps in disease pathogenesis [47, 48]. LL37 also binds dsRNA and activates a MAVS pathway in keratinocytes, which initiates a type I interferon response [30] and LL37/Poly(I:C) complexes were shown to activate RIG-I in oral keratinocytes [31]. Neutrophil extracellular trap-associated RNA, pre-bound to LL37 in resting neutrophils, when released activates NOD2 signalling in keratinocytes [49]. LL37-facilitated uptake of nucleic acid is important for alerting neighbouring cells to danger and, as we demonstrate here, this results in the release of IL-36γ. We observed no upregulation of IL-36α, demonstrating selectivity in this subfamily of cytokines. In addition to IL-36γ release, LL37/dsRNA complexes also induce CXCL10 production. Interestingly, a role for CXCL10 in psoriasis pathogenesis has been proposed and CXCL10 levels in patient serum has been suggested as a potential biomarker of disease severity [50, 51]. This represents a more physiologically restrained immune response than transfection of Poly(I:C) and indeed IL-36γ was not processed to its more potent mature form as keratinocyte proteases remain compartmentalised in the absence of cell death. In this context, neutrophil infiltration and secretion of proteases are required to unleash potent IL-36γ activity, as in the pathogenesis of GPP.

Expression of IL-36γ is lost in *ASC*- and *GSDMD*-deficient N/TERT-1 cells in response to LL37/Poly(I:C) complexes. In primed N/TERT-1 cells, GSDMD is dispensable for IL-36γ release in response to LL37/Poly(I:C) complexes, indicating that IL-36γ expression is also induced in a positive feedback loop, probably by the release of IL-1 family cytokines through GSDMD pores. Previously, IL-1β was shown to be a strong activator of IL-36γ mRNA expression in keratinocytes [52]. This demonstrates another key difference between IL-36γ and IL-1β, the former requires upregulation of expression, while in human keratinocytes IL-1β and NLRP1 are constitutively expressed.

A number of studies have shown increased IL-36γ transcript levels in response to IL-17A, TNFα and IL-22, with these cytokines often showing synergistic effects in keratinocytes [14, 53]. Moreover, Mercurio and colleagues showed release of IL-36 cytokines from HPKs in response to IL-17A particularly in synergy with TNFα and IFNγ [54]. In our hands, IL-17A stimulation fails to induce IL-36γ release, however, together with IFNγ we observed a slight but insignificant increase after 8 h co-stimulation. Additionally, it was reported that ATP enhanced IL-1α and TNFα-induced IL-36γ secretion from HPKs [14]. Since ATP can activate the NLRP3 inflammasome in keratinocytes [55] this might be why more IL-36γ is released in this manner.

In addition to IL-36γ expression requiring priming, responsiveness to LL37/Poly(I:C) complexes is also accelerated when cells are co-treated with IFNγ, suggesting that the LL37/Poly(I:C) sensor is lowly expressed at basal levels, unlike NLRP1 and Protein Kinase R (PKR) [56]. Indeed, RIG-I and MDA5 expression levels are enhanced upon IFNγ treatment and stimulation of primary keratinocytes with a RIG-I specific agonist was sufficient to induce IL-36γ release. Our results show that sensing of LL37/dsRNA through RLR-pathways is central to IL-36γ release from keratinocytes, which has physiological relevance to psoriatic disease pathogenesis. As mentioned, RIG-I and MDA5 have been discovered as risk alleles for psoriasis vulgaris [24, 25]. RIG-I-deficient mice are protected against IL-23- and imiquimod-induced psoriasiform disease [57] and injection of RIG-I ligand 5′ppp-dsRNA to the ear of wild-type mice facilitates IL-23-mediated psoriasis-like skin inflammation in mice [57]. One symptom that can be associated with Singleton-Merten syndrome, is the development of psoriatic skin lesions [58] and a RIG-I gain-of-function mouse that harbours a Singleton-Merten syndrome-associated mutation, develops psoriasis-like lesions on the tail and back, suggesting that in this model areas of mechanical stress might be more prone to the development of lesions [59]. In human psoriasis, the Koebner phenomenon can result in new psoriatic lesions at sites of skin injury, which involves the upregulation of LL37 and the release of nucleic acids from damaged keratinocytes [30]. Here, we demonstrate that these danger signals can activate RLRs and MAVS-dependent release of IL-36γ in keratinocytes. MAVS enables Caspase-3 activation and subsequent GSDME cleavage. Interestingly, Guy and colleagues recently described that PKR can induce GSDME activation [56], which was independent of MAVS, suggesting that GSDME-dependent pyroptosis might be a potential outcome common to cytoplasmic nucleic sensor activation. Interestingly, the 2A2 protein of duck hepatitis A virus type 1 interacts with MAVS and can induce GSDME-mediated pyroptosis [60].

The role of the cleaved Caspase-3 and GSDME to psoriasis pathogenesis merits further exploration. Full-length Caspase-3 expression was found to be increased in the epidermis of psoriasis patients in an Egyptian cohort [61] and GSDME-deficient mice are protected against imiquimod-induced psoriasiform disease [62]. Barrier function is important at the epidermis and primary keratinocytes might initiate a mechanism by which to repair or recycle GSDME pores to sustain barrier integrity. Pore repair by the ESCRT machinery might be a means in which primary keratinocytes are protected from pyroptosis, as previously described in macrophages for GSDMD [63]. Notably, human primary keratinocytes are less susceptible to cell death associated with LL37/Poly(I:C)-induced GSDME pore formation than CRISPR/Cas9-modified N/TERT-1 cells and it is of interest to further explore the mechanisms of pore repair in these cells.

While IL-36 cytokines directly contribute to the pathogenesis of psoriasis vulgaris and pustular psoriasis in the skin and the significance of therapeutically targeting IL-36 cytokines has been shown by Bachelez and colleagues who demonstrated the effectiveness of utilising a specific monoclonal antibody against the IL-36R in GPP patients harbouring or lacking *IL36RN* mutations [64]. Understanding the mechanisms controlling IL-36 cytokine release might also be beneficial to target specific cytokines of the IL-36 family in inflammatory diseases. Much remains to be dissected concerning the biology and regulation of the IL-1 cytokine superfamily in barrier tissue and of IL-36 cytokines specifically.

## Materials and methods

### Cell culture

HEK293 T cells were cultured in Dulbecco’s modified Eagle’s medium containing 4.5 g/L glucose (DMEM, Gibco, Paisley, Scotland) supplemented with 10% (v/v) foetal bovine serum (FBS, Biowest, Nuaillé, France), and Antibiotic-Antimycotic solution (100X, Gibco). Human primary keratinocytes (HPKs) and N/TERT1 cells were cultured in Keratinocyte Serum Free Medium (KSFM, Gibco), supplemented with epithelial growth factor and bovine pituitary extract (EGF and BPE, Gibco). HPKs were isolated as described previously [65] or were purchased from ATCC (Manassas, VA). Aliquots were stored at liquid nitrogen and upon thawing were resuscitated in complete medium (KSFM with supplements) in a T75 culture flask. Upon reaching 80% confluency, HPKs were expanded into T175 flasks and when 70–80% confluency were seeded for experiments. All cells were maintained at 37 °C in a humidified atmosphere of 5% CO_2_.

### Keratinocyte stimulation

HPKs and N/TERT-1 were seeded at 1 × 10^5^ cells/ml in supplemented medium and treated when 70–80% confluent. Supplement-free medium was used for mock treatment or preparation of the individual treatments. For transfection, Lipofectamine 2000 (Lipo, Invitrogen, Waltham, MA, USA) was pre-incubated in supplement-free medium in a 1:1 ratio with 0.01–1 μg/ml high molecular weight (HMW) Poly(I:C), 1 μg/ml Poly(dA:dT) or 1 μg/ml 5’ triphosphate hairpin RNA (all Invivogen, San Diego, CA, USA). Alternatively, 1–5 μg/ml Poly(I:C) HMW, Poly(dA:dT) or 5’ triphosphate hairpin RNA (hpRNA) was complexed in a 1:1 ratio with 1–5 μg/ml LL37 (Invivogen) for 15 min, before dilution with supplement-free medium and addition to cells. The same concentrations were used for corresponding single treatments and KSFM without supplements was used at the corresponding volume for mock treatments unless otherwise stated in the figure legend. Further treatments used include Val-boroPro (VbP, 0.5–3 μM Lucerna-Chem, Lucerne, Switzerland), anisomycin (ANS, 1 μM, Sigma-Aldrich, St. Louis, MO, USA) or DMSO and a single dose of ultraviolet B irradiation (UVB, 0.0875 J/cm^2^). Cells were incubated with treatments as outlined in the figure legends. In priming experiments, cells were incubated for indicated time-points with IL-17 A (10 or 100 ng/ml, PeproTech, Cranbury, NJ, USA), TNF-α (5 ng/ml, PeproTech) or IFNγ (10 ng/ml, PeproTech). Treatments were diluted in supplement-free KSFM medium, so non-primed cells were treated with medium only. To inhibit activity of downstream kinases and caspases, HPKs were treated with the inhibitors Belnacasan (Caspase-1 inhibitor, 10 µM, MedChemExpress, South Brunswick, NJ, USA) or SB-203580 (p38 MAPK inhibitor, 20 µM, Selleckchem, Houston, TX, USA), MCC950 (NLRP3 inhibitor, 5 µM, Selleckchem, Houston, TX, USA) or DMSO control 1 h before treatment as described above.

HEK293 T cells or HEK293^IL-36γ^ cells were seeded (2 × 10^5^ cells/ml; 3 ml) in six-well plates and transfected, using Lipofectamine 2000, with pcDNA3.1 plasmids encoding MAVS (#52000, Addgene), Caspase-3 (#11813, Addgene), GSDME (#154876, Addgene, sub-cloned into pcDNA3.1) or empty vector as described in figure legends (2 µg total DNA, 1:1 ratio). Transfected cells were incubated at 37 °C for 24 h.

Cells were incubated 30–60 min with Propidium Iodide (PI, 1 µg/ml, Sigma-Aldrich) and Hoechst 33258 (Hoechst, 10 µg/ml, Thermo Fisher Scientific, Waltham, MA USA). Cells were then imaged with the Cytation 3 (Biotek, Winooski, VT, USA). The analysis of PI and Hoechst double resp. single positive nuclei was performed with Gen5 Image+ 3.12 software and the results are shown as the percentage of PI^+^ Hoechst^+^ double positive cells from all Hoechst^+^ positive cells.

### Measurement of cytotoxicity by lactate dehydrogenase (LDH) assay

Cell lysis was assessed by measuring the release of lactate dehydrogenase (LDH) from supernatants of HPK and N/TERT-1 cells. These were collected at indicated time points, the total LDH content of cells was determined by lysing in medium containing 5% Triton X-100 for 30 min at 37 °C. Samples were centrifuged to pellet cells and discard cell-debris, and subsequently LDH activity was measured in supernatants in duplicate for each sample using the CytoTox 96® Non-Radioactive Cytotoxicity Assay (Promega, Madison, WI, USA) according to the manufacturer’s instructions. Absorbance was measured with the Cytation 3 (Biotek) or Spark 10 M (Tecan, Männedorf, Switzerland) plate reader, values of treated samples were compared to the mean total LDH content, and are shown as percentage release.

### Immunoblotting

Cells were washed in ice-cold PBS, scraped into tubes and spun for 5 min at 8000 × *g*, and lysed for 20 min in ice-cold lysis buffer containing 1x phosphatase inhibitor (Bimake, Houston, TX, USA) and 1x protease inhibitor (Bimake). Lysates were then centrifuged at 13,000 × *g* at 4 °C for 10 min. Protein content was determined by Pierce™ BCA Protein Assay Kit (Thermo Fisher Scientific). Lysates were diluted with equal amount of SDS-loading buffer, incubated at RT for 10 min, and boiled for 5 min at 95 °C. Cell culture supernatants were precipitated by adding 4 volumes of acetone (100% v/v), incubating overnight at −20 °C and centrifugation for 90 min (3200 × *g* at 4 °C). The protein pellet was resuspended in SDS-loading buffer, sonicated and boiled for 5 min. Equal amounts of proteins were separated by SDS-PAGE, transferred to nitrocellulose and membranes were probed with indicated antibodies and incubated rocking overnight at 4 °C. After washing, membranes were incubated for 1 h at room temperature with indicated alkaline phosphatase-conjugated secondary antibodies in milk, and protein signal was visualised by BCIP/NBT Colour Development Substrate (Promega).

### Biotin-labelled co-immunoprecipitation

HPKs were transfected by the 1 μg/ml HMW Poly(I:C) coupled with biotin (Invivogen) using Lipofectamine 2000, or treated with 1 μg/ml LL37 with and without pre-incubation with 1 μg/ml HMW Poly(I:C) coupled with Biotin (Invivogen). Cells were harvested as described above and incubated overnight at 4 °C with Strepavidin beads (Dynabeads, 5% V/V Thermo Fisher Scientific). Beads were isolated magnetically and washed four times with lysis buffer. The beads were resuspended in SDS loading buffer, incubated for 10 min at RT, boiled at 95 °C for 5 min and subjected to immunoblotting with the indicated primary and secondary antibodies.

### Triton X-100 insoluble fractionation of keratinocytes

Cells were lysed in a Triton X-100-containing buffer (0.5% Triton X-100 in 50 mM Tris/HCl, pH 7.6) containing phosphatase and protease inhibitors. Lysates were centrifuged for 15 min at 6000 × *g*. After centrifugation, supernatants (Triton X-soluble fractions) were quantified for amount of protein and stored at −20 °C. Pellets (Triton X-100-insoluble fractions) were washed twice with 1X TBS and resuspended in 500 μl TBS. The resuspended pellets were cross-linked for 45 min at 37 °C with 2 mM disuccinimidyl suberate (Pierce) and then centrifuged for 15 min at 6000 × *g*. The pellets were resuspended in SDS-loading buffer, sonicated, boiled at 95 °C for 5 min. Both fractions were analysed by SDS-PAGE and immunoblotting with the indicated primary and secondary antibodies.

### Generation of CRISPR/Cas9 knock out cell lines

sgRNAs were designed using the Benchling platform (https://www.benchling.com), and single-stranded DNA oligonucleotides (Microsynth, Balgach, Switzerland) were cloned into the lentiCRISPRv2 plasmid (#52961, Addgene). Plasmids were co-transfected by Lipofectamine 2000 into HEK293 T cells with the packaging vectors psPAX2 and pMD2.G (#12260 and #12259, Addgene), and after 48 h lentiviruses were harvested and filtered through 0.45 μm Syringe-filters (Millipore, Burlington, MA, USA). CRISPR/Cas9 knock out cell lines were created in N/TERT1 cells by lentiviral transduction. 24 h after plating, cells were transduced in the presence of 2.5 μg/ml polybrene (hexadimethrine bromide) (Sigma-Aldrich). 6 h after transduction, medium was replaced with supplemented KSFM. After another 24 h, medium was changed to medium containing puromycin (5 μg/ml) for selection. Single cell colonies were created by plating the cells at a density of 1 cell/200 μl medium on 96 well-plates and maintained individually. Knock-out efficiency was analysed by sequencing and immunoblotting.

### Generation of stable cell lines

For generating HEK 293T cells expressing IL-36γ, a pLX304 lentiviral plasmid encoding human full-length V5-tagged IL-36γ (DNASU, Arizona State University, USA) was packaged into lentivirus by transfecting the vector (1.9 μg) along with psPAX2 (750 ng), and pMD2.G (250 ng) using Lipofectamine transfection reagent (Promega) into HEK 293T cells and after 48 h lentiviruses were harvested and filtered through 0.45 μm Syringe-filters (Millipore, Burlington, MA, USA) and then used to infect HEK293 T cells. After 48 h, the cells expressing IL-36γ were selected with blasticidin (10 μg/mL).

### Genomic DNA isolation, PCR and sequencing

Genomic DNA from cells was isolated by the QiAMP DNA Blood Minikit (Qiagen, Venlo, Netherlands) and genomic regions of interest were amplified by PCR on the Thermocycler (Labgene Scientific SA, Châtel-St-Denis, Switzerland) using specific primers, followed by DNA purification with kit (New England Biolabs, Ipswich, MA, USA) and sequencing (Microsynth).

### Immunofluorescent staining

Primary human keratinocytes were seeded directly on coverslips and treated as described above, treated with fluorescein-labelled HMW Poly(I:C) incubated with LL37 (1:1). After the indicated time-points, cells were gently washed three times in PBS. Cells were then fixed by the addition of 4% paraformaldehyde for 30 min at room temperature or overnight at 4 °C. Cells were washed and permeabilised for 2 min with 0.2% Triton X-100 in PBS. Cells were washed, blocked, and incubated with indicated primary antibodies in 3% BSA in PBST overnight at 4 °C. Cells were washed and incubated for 1 h at room temperature with the indicated secondary antibodies and DAPI in 3% BSA in PBS. Cells were washed and coverslips were mounted onto a slide with ProLong® Gold anti-fade reagent (Molecular Probes, Eugene, OR, USA). Confocal images were captured using the ×63 objective lens on the Leica TCS SP5 laser-scanning microscope equipped with the appropriate filter sets.

### RNA isolation and real-time RT-PCR

Total RNA was extracted from HPKs using Tri-Reagent (Sigma-Aldrich) and quantified using a Nanodrop (ACTGene, LabGene Scientific). cDNA was generated from RNA using the RevertAid First Strand cDNA Synthesis Kit (Thermo Fisher Scientific). Quantitative real-time PCR was performed in duplicates for each sample using a LightCycler®480 (Roche, Basel, Switzerland) with SYBR Green I Master mix (Roche) and specific primers. The mean Ct value of the gene of interest was normalized to the expression of the RPL27 housekeeper, followed by normalization of the treated sample to the mock sample from the same donor.

### ELISA

Cell culture supernatant was collected from cells at indicated time-points after treatment and cell debris was removed by centrifugation at 5000 × *g* for 5 min at 4 °C. Levels of IL-1β (R&D Systems), IL-36γ (R&D Systems), and CXCL10 (Biolegend) were measured in duplicates for each sample by ELISA according to the manufacturer’s instructions and absorbance readings were performed on the Cytation 3 (Biotek) or Spark 10 M (Tecan) plate readers. The protein concentration was calculated from the mean absorbance of the sample with an 8-point linear standard curve.

### Statistical analysis

Data was visualised and statistically analysed using GraphPad Prism 9 software (GraphPad Software, La Jolla, CA, USA). The statistical test used is indicated in the corresponding figure legend. Differences were considered significant when *p < 0.05, **p < 0.01, ***p < 0.001, ****p < 0.0001.

## Supplementary information

Supplementary Tables and Figures

Uncropped blots

## Data Availability

The authors declare that all data supporting the findings of this study are available within the article and the Supplementary Information. All other data are available from the corresponding author upon request.
