# Supplementary material for: LL37 complexed to double-stranded RNA induces RIG-I-like receptor signalling and Gasdermin E activation facilitating IL-36γ release from keratinocytes
Source: Cell Death Dis. 2025 Mar 22;16(1):198. doi: 10.1038/s41419-025-07537-9 (PMC11929817; doi:10.1038/s41419-025-07537-9)
Supplement: Supplementary file 1 — Supplementary Tables and Figures [file 41419_2025_7537_MOESM1_ESM.docx]

**Table S1. Single-guide RNA sequences used in this study**

| Target | Forward 5’-3’ | Reverse 5’-3’ |
| --- | --- | --- |
| Gasdermin D (sgRNA) | CACCGAATGTGTACTCGCTGAGTG | AAACCACTCAGCGAGTACACATTC |
| Gasdermin E (sgRNA) | CACCGATGAAGACTGGCTCTCTACG | AAACCGTAGAGAGCCAGTCTTCATC |
| MAVS (sgRNA) | CACCGCTGGTAGCTCTGGTAGACAG | AAACCTGTCTACCAGAGCTACCAGC |
| MDA5 (sgRNA) | CACCGCGTCTTGGATAAGTGCATGG | AAACCCATGCACTTATCCAAGACGC |
| PYCARD (ASC) (sgRNA) | CACCGAACTTCTTGAGCTCCTCGG | AAACCCGAGGAGCTCAAGAAGTTC |
| RIG-I (DDX58) (sgRNA) | CACCGTTCCTGTTGGAGCTCCAGG | AAACCCTGGAGCTCCAACAGGAAC |

**Table S2. Oligonucleotides used in this study**

| Target | Forward 5’-3’ | Reverse 5’-3’ |
| --- | --- | --- |
| *ISG15* (qPCR primer) | GCGCAGATCACCCAGAAGAT | TCCTCACCAGGATGCTCAGA |
| *IFNβ (IFNb)* (qPCR primer) | GCTTGGATTCCTACAAAGAAGCA | ATAGATGGTCAATGCGGCGTC |
| *IL-36γ (IL1F9)* (qPCR primer) | AGGAAGGGCCGTCTATCAATC | CACTGTCACTTCGTGGAACTG |
| *RPL27* (qPCR primer) | ATCGCCAAGAGATCAAAGATAA | TCTGAAGAATC CTTATTGACG |

**Table S3. Antibodies used in this study**

| ANTIBODY | VENDOR | CATALOG NUMBER |
| --- | --- | --- |
| ASC (for Western Blot) | Adipogen | 210-905 |
| ASC (for Immunofluorescence) | Santa Cruz | sc-22514-R |
| β-actin | Sigma-Aldrich | A5441 |
| Caspase-7 | Abcam | 201959 |
| Caspase-8 | Cell Signaling Technology | 9746 |
| Cleaved Caspase-1 | Santa Cruz | sc-622 |
| Cleaved Caspase-3 | Cell Signaling Technology | 9661L |
| Cathepsin S | Santa Cruz | sc-271619 |
| Gasdermin D | Novus Biologicals | NBP2-33422 |
| Gasdermin E | Cell Signaling Technology | 84005S |
| Gasdermin E | Proteintech | 13075-1-AP |
| IgG anti-rabbit Isotype control | Abcam | ab172730 |
| IL-36γ | R&D Systems | AF2320 |
| IL-1β | R&D Systems | MAB201 |
| MAVS | Cell Signaling Technology | 3993T |
| MDA5 | Cell Signaling Technology | 5321 |
| NLRP1 | Biolegend | 679802 |
| p38 MAPK | Cell Signaling Technology | 8690 |
| p-p38 MAPK | Cell Signaling Technology | 4511 |
| RIG-I | Cell Signaling Technology | 3743 |
| TBK1 | Cell Signaling Technology | 3504 |
| p-TBK1 | Cell Signaling Technology | 5483 |
| anti rabbit igG Dye light 488 | Abcam | ab96883 |
| anti-goat IgG (H+L)-AP | BIO-RAD | 1721037 |
| anti-mouse IgG-AP | Promega | S3721 |
| anti-rabbit IgG-AP | Promega | S3731 |


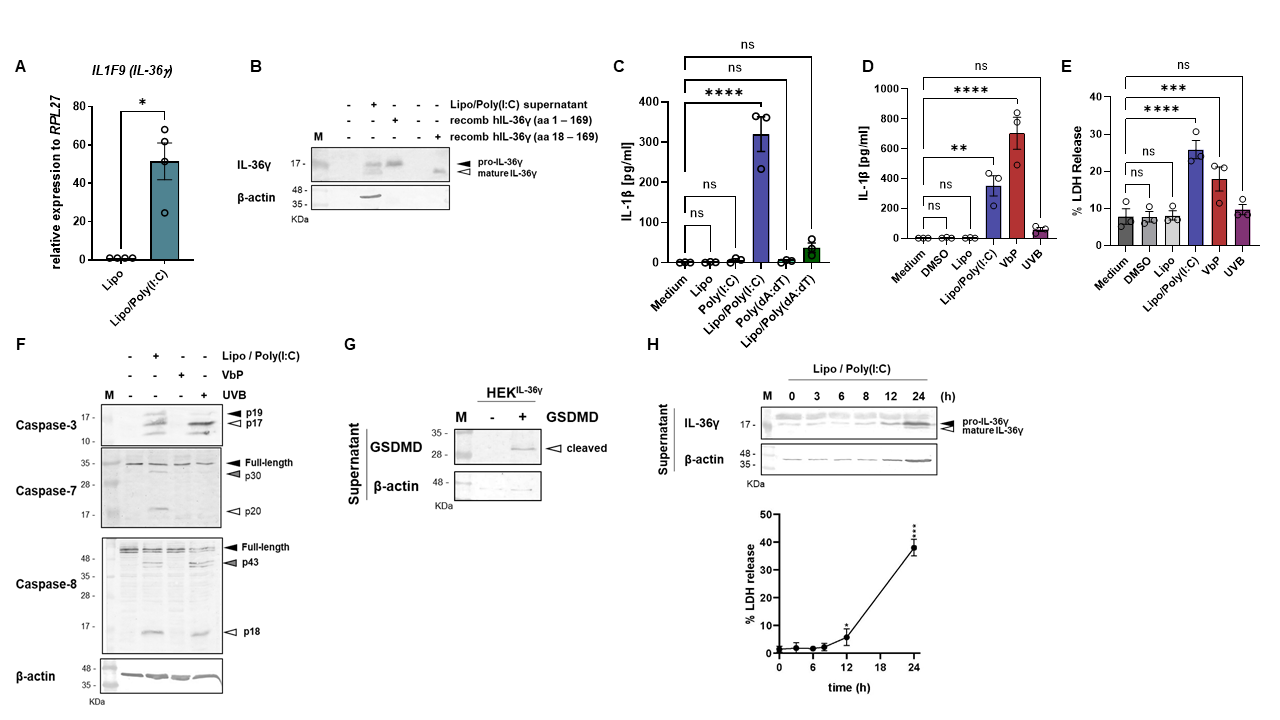


**Supplementary Figure 1 Intracellular dsRNA is a potent inducer of IL-36γ release from human primary keratinocytes**

(**A**) HPKs were transfected with Poly(I:C) (1 μg/ml) for 6 h. RNA was extracted and mRNA was subjected to qPCR analysis for expression of *IL1F9 (IL-36γ)*. (**B**) HPKs were transfected with Poly(I:C) (1 μg/ml) for 24 h, supernatants were subjected to SDS-PAGE alongside full length and mature human recombinant IL-36γ proteins (amino acids 1-169 and 18-169, respectively). (**C**) HPKs were transfected with Poly(I:C) or Poly(dA:dT) (1 μg/ml) for 24 h and IL-1β levels were measured by ELISA. (**D - E**) HPKs were transfected with Poly(I:C) (1 μg/ml), stimulated with VbP (1 μM), one UVB pulse (0.0875 J/cm^2^), DMSO or Lipo, (1 μg/ml) for 24 h. Supernatants were assayed for (**D**) IL-1β release by ELISA or (**E**) LDH assay. (**F**) HPKs were transfected with Poly(I:C) (1 μg/ml), stimulated with VbP (3 μM), one UVB pulse (0.0875 J/cm^2^) or medium for 24 h and cell lysates were subjected to SDS-PAGE and immunoblotting with indicated antibodies. (**G**) HEK293^IL-36γ^ cells were transfected with GSDMD or empty vector (EV) (1 μg) for 24 h. Supernatants were subjected to SDS-PAGE and immunoblotting. (**H**) HPKs were transfected with Poly(I:C) (1 μg/ml) and supernatants were collected at indicated time-points. Supernatants were subjected to LDH assay (bottom panel) and SDS-PAGE followed by immunoblotting with anti-IL-36γ and β-actin antibodies. Data are presented as (**B, F - H**) representative of 3 independent experiments or presented as the mean ±S.E.M. of 3 (**E, F, H**) or 4 (**A, C**) independent experiments and analysed with one-way ANOVA followed by Dunnett’s multiple comparisons test or (**A**) a two-tailed t test. *p<0.05, ***p<0.001, ****p<0.0001. ns = non-significant. Lipo = Lipofectamine 2000, M = protein marker.


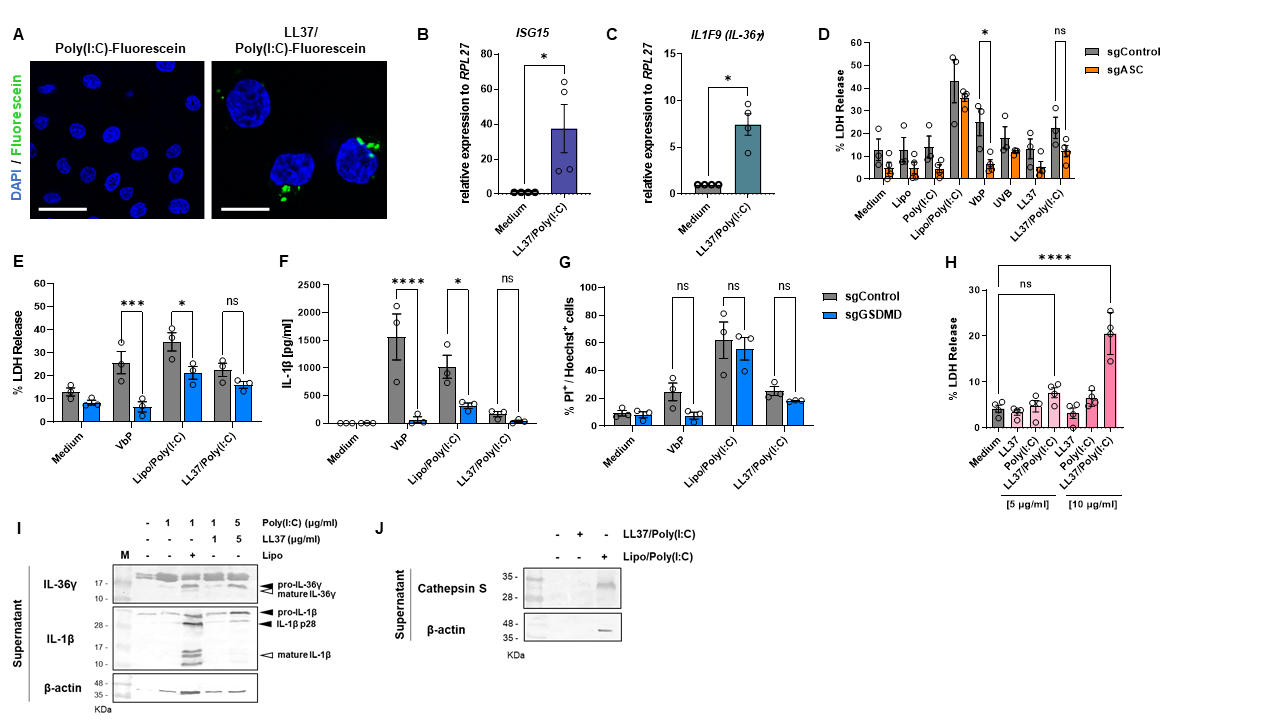


**Supplementary Figure 2 LL37/Poly(I:C) complexes induce IL-36γ release form HPKs in the absence of cell death**

**(A)** HPKs were stimulated with Fluorescein-labelled Poly(I:C) and LL37 complexes (1:1 μg/ml) for 24 h. Cells were visualized for Fluorescein-labelled Poly(I:C) by confocal microscopy. Nuclei were stained with DAPI. Scale bar = 30 μm (left panel) and 10 μm (right panel). (**B, C**) HPKs were stimulated with LL37/Poly(I:C) complexes for 6 h. RNA was extracted and mRNA was subjected to qPCR analysis for expression of (**B**) *ISG15 and (****C****) IL1F9 (IL-36γ)*. (**D**) *ASC-*deficient N/TERT-1 cells (sgASC) were transfected with Poly(I:C) (1 μg/ml), stimulated with LL37/Poly(I:C) (5:5 μg/ml) complexes, VbP (0.5 μM), or one UVB pulse (0.0875 J/cm^2^) for 18 h and supernatants were subjected to LDH assay. (**E - G**) *GSDMD-*deficient N/TERT-1 cells (sgGSDMD) were transfected with Poly(I:C) (1 μg/ml), stimulated with LL37/Poly(I:C) (5:5 μg/ml) complexes or VbP (1 μM) for 18 h and (**E, G**) supernatants were analysed for (**E**) LDH and (**F**) IL-1β levels or (**G**) cells were assessed for PI positivity. (**H**) HPKs were stimulated with LL37/Poly(I:C) (5:5 μg/ml or 10:10 μg/ml) complexes for 24 h, supernatants were analysed for LDH release. (**I, J**) HPKs were stimulated with LL37/Poly(I:C) complexes (**I**) (1 or 5 μg/ml) (**J**) (5 μg/ml), transfected with Poly(I:C) (1 μg/ml) or medium (-) for 24 h. Supernatants were subjected to SDS-PAGE and immunoblotting with indicated antibodies. Data are presented as a representative (**A, I, J**) of three independent experiments or are presented (**B - H**) as the mean ±S.E.M. of 3 or 4 independent experiments and subjected to (**B, C**) a two-tailed t test, (**D-G**) two-way ANOVA followed by Šidák’s multiple comparisons test or (**H**) one-way ANOVA followed by Dunnett’s multiple comparisons test. *p<0.05, **p<0.01, ***p<0.001, ****p<0.0001. ns = non-significant. Lipo = Lipofectamine 2000, M = protein marker, sg = single-guide RNA.


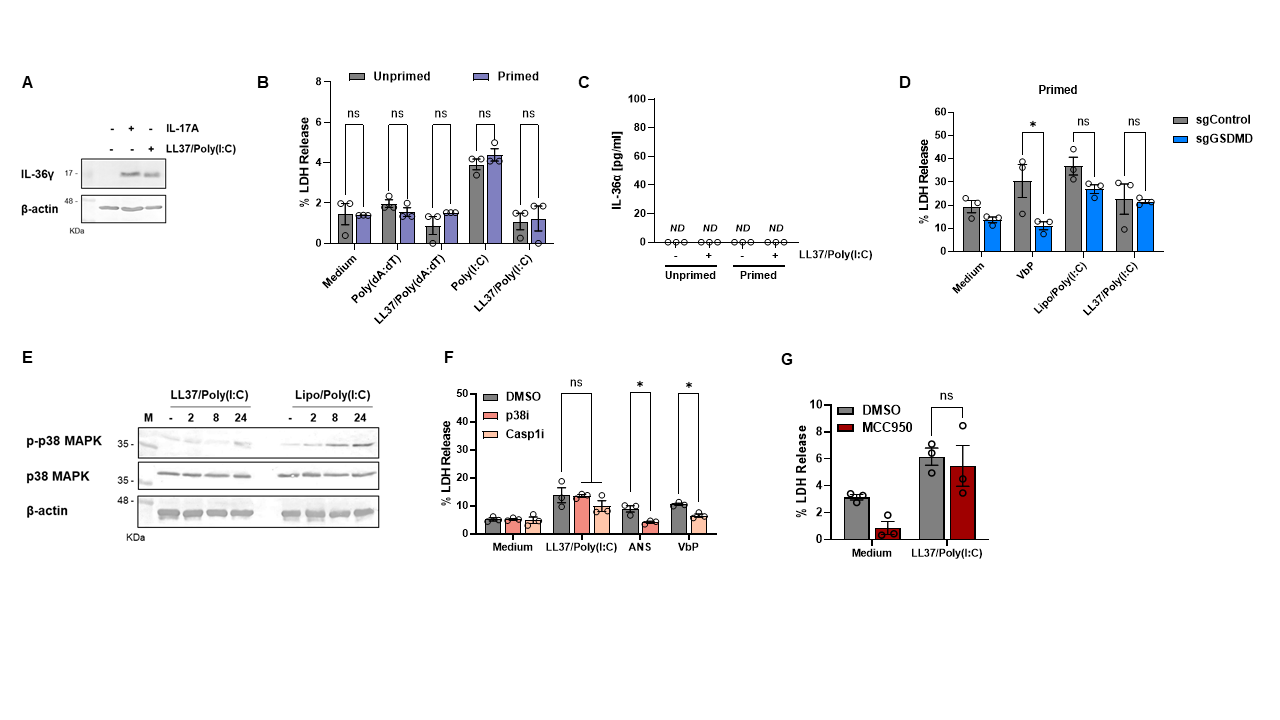


**Supplementary Figure 3 LL37/Poly(I:C) complexes induce IL-36γ release from keratinocytes independent of inflammasome and GSDMD activity**

(**A**) HPKs were stimulated with IL-17A (100 ng/ml), LL37/Poly(I:C) complexes or medium for 18 h. Cell lysates were subjected to immunoblotting. (**B**) HPKs were primed with IL-17A (100 ng/ml) left unprimed for 6 h prior to stimulation with Poly(I:C), Poly(dA:dT), LL37/Poly(I:C) or LL37/Poly(dA:dT) complexes (all 5 μg/ml) for 18 h. Supernatants were analysed for LDH release. (**C**) HPKs were primed with IL-17A (100 ng/ml) for 6 h or left unprimed prior to stimulation with LL37/Poly(I:C) complexes (5 μg/ml) or medium (-) for 18 h. Supernatants were analysed by ELISA for IL-36α levels. (**D**) *GSDMD-*deficient N/TERT-1 cells (sgGSDMD) were stimulated with IL-17A (100 ng/ml) for 6 h prior to stimulation with Lipo/Poly(I:C) (1 μg/ml), LL37/Poly(I:C) complexes (5 μg/ml) and VbP (1 μM) for 18 h. Supernatants were analysed for LDH release. (**E**) HPKs were stimulated with LL37/Poly(I:C) complexes (5 μg/ml) or were transfected with Poly(I:C) (1 μg/ml) for indicated times (control treatment (-) indicates medium or Lipo, respectively). Cell lysates were subjected to SDS-PAGE and immunoblotting with indicated antibodies. (**F**) HPKs were primed with IL-17A (10 ng/ml) and TNF-α (5 ng/ml) for 5 h, followed by inhibitor treatment with SB 203580 (p38i, 20 μM), Belnacasan (Casp1i, 10 μM), or DMSO for 1 h before stimulation with LL37/Poly(I:C) complexes (5 μg/ml), VbP (1 μM), or Anisomycin (ANS, 1 μM) for 18 h. Supernatants were measured for the release of LDH. (**G**) HPKs were primed with IL-17A for 6 h prior to stimulation with LL37/Poly(I:C) complexes for 18 h. Cells were treated with MCC950 1 h before stimulation. Supernatants were measured for LDH release. Data are presented as a representative (**A, E**) of three independent experiments or are presented (**B - D, F - G**) as the mean ±S.E.M. of 3 independent experiments and subjected to (**B - D, G**) two-way ANOVA followed by Šidák’s or (**F**) two-way ANOVA followed by Dunnett’s multiple comparisons test. *p<0.05. ns = non-significant. Lipo = Lipofectamine 2000, M = protein marker, ND = not detectable, sg = single-guide RNA.


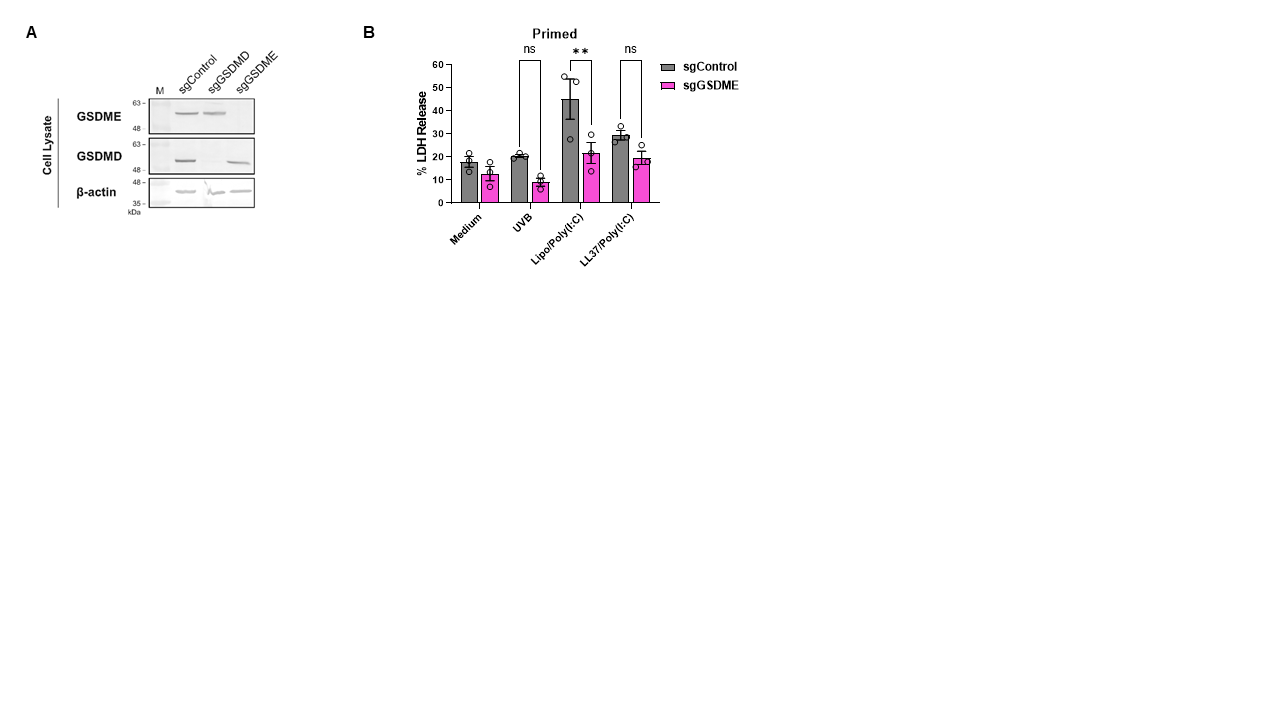


**Supplementary Figure 4 LL37/Poly(I:C) complexes activate GSDME cleavage in keratinocytes**

(**A**) Cell lysates from Control and *GSDMD-* and *GSDME-*deficient N/TERT-1 cell-lines (sgControl / sgGSDMD / sgGSDME) were subjected to SDS-PAGE and immunoblotting with indicated antibodies. (**B**) *GSDME-*deficient N/TERT-1 cells (sgGSDME) were primed with IL-17A (100 ng/ml) for 6 h prior to stimulation with Lipo/Poly(I:C) (1 μg/ml), LL37/Poly(I:C) complexes (5 μg/ml) or one UVB pulse (0.0875 J/cm^2^) for 18 h. Supernatants were analysed for LDH levels. Data are presented as (**B**) the mean ±S.E.M. of 3 independent experiments and subjected to two-way ANOVA followed by Šidák’s or multiple comparisons test. **p<0.01. ns = non-significant. Lipo = Lipofectamine 2000, M = protein marker, sg = single-guide RNA.


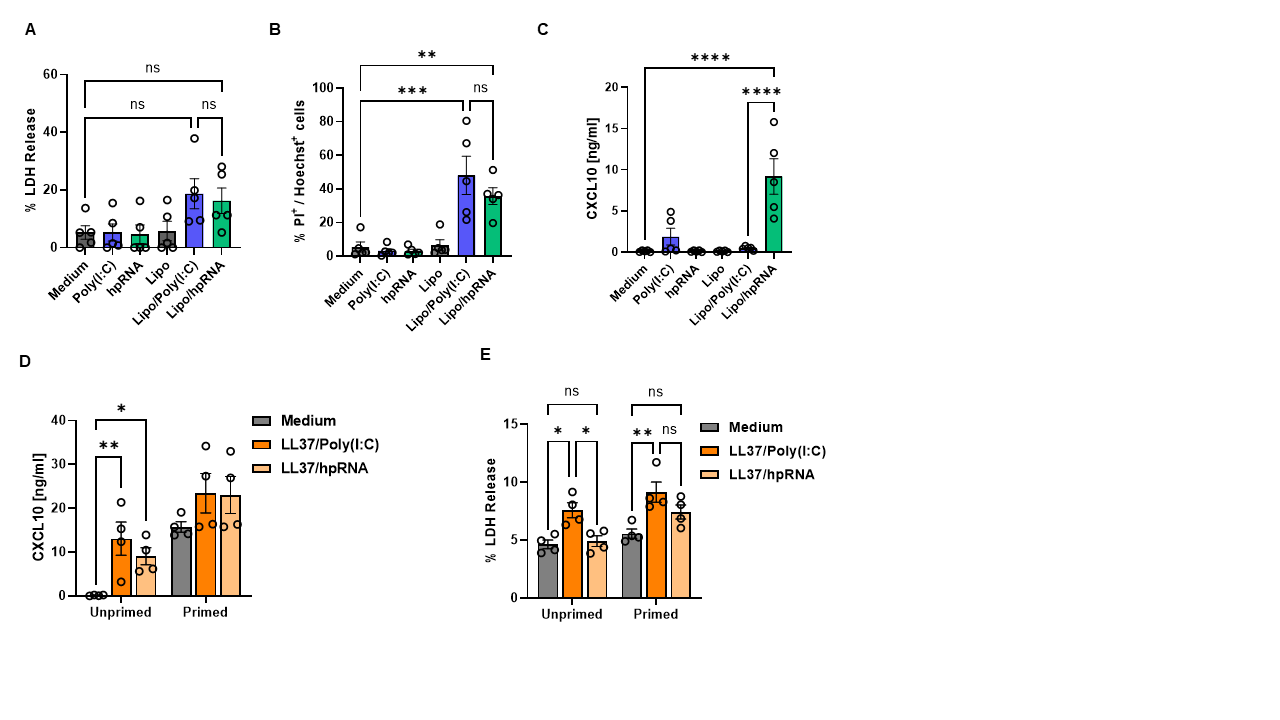


**Supplementary Figure 5 RIG-I-like Receptor activation mediates IL-36γ release in human primary keratinocytes**

(**A - C**) HPKs were primed with IL-17A (100 ng/ml) for 6 h prior to liposomal transfection of Poly(I:C) (1 μg/ml) or hpRNA (1 μg/ml) for 18 h. (**A, C**) Supernatants were analysed for (**A**) LDH or (**C**) CXCL10 release or (**B**) cells were assessed for PI positivity. (**D, E**) HPKs were primed with IL-17A (100 ng/ml) and IFNγ (10 ng/ml) or left unprimed for 16 h, followed by stimulation with LL37/Poly(I:C) or LL37/hpRNA complexes (both 5 μg/ml) for 8 h. Supernatants were subjected to (**D**) ELISA to measure CXCL10 levels or (**E**) LDH assay. Data are presented as the mean ±S.E.M. of at least 4 independent experiments and subjected to two-way ANOVA followed by Šidák’s multiple comparisons test. *p<0.05, **p<0.01, ***p<0.001, ****p<0.0001. ns = non-significant. Lipo = Lipofectamine 2000, M = protein marker.


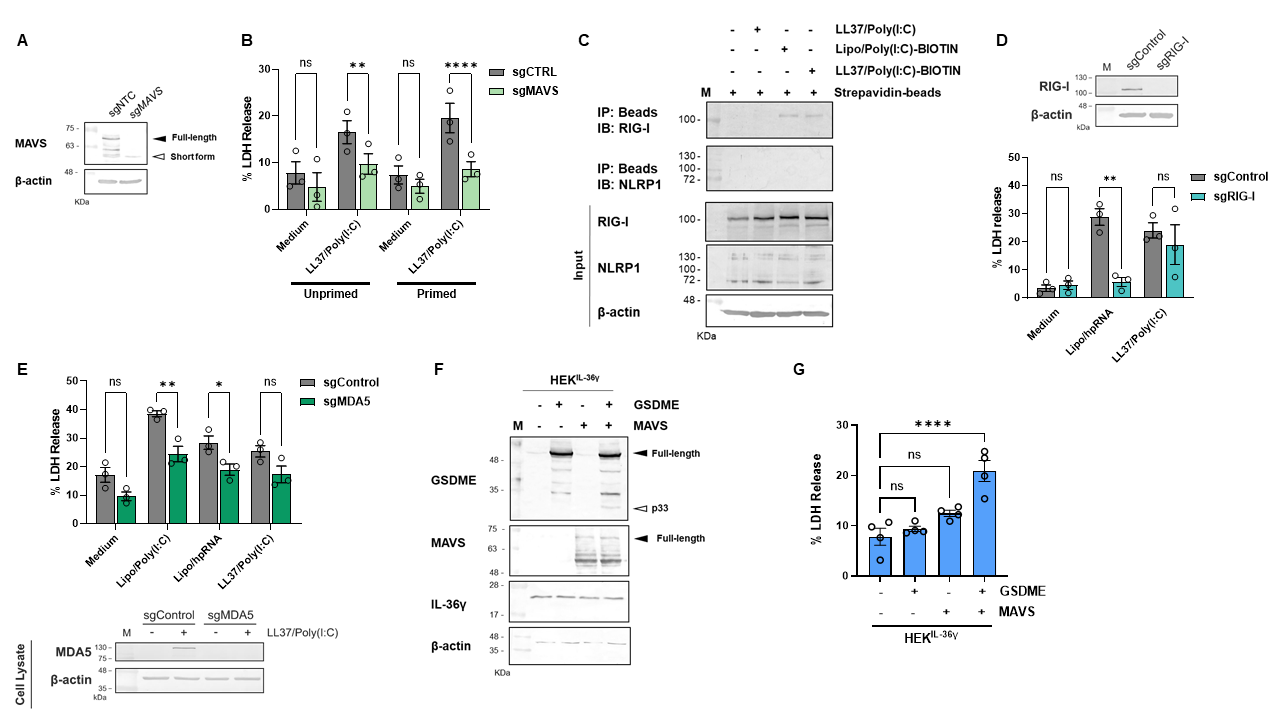


**Supplementary Figure 6 Activation of the RLR/MAVS pathway mediates LL37/Poly(I:C)-induced IL-36γ release from human primary keratinocytes**

(**A**) Cell lysates from Control and *MAVS-*deficient N/TERT-1 cell-lines (sgNTC / sgMAVS) were subjected to SDS-PAGE and immunoblotting with indicated antibodies. (**B**) *MAVS-* (**D**) *RIG-I* or (**E**) *MDA5-*deficient N/TERT-1 cell-lines (sgMAVS / sgRIG-I / sgMDA5) were primed with IL-17A (100 ng/ml) for 6 h prior to treatment with LL37/Poly(I:C) complexes (5 μg/ml) or transfection with either Poly(I:C) or hpRNA (both 1 μg/ml) for 18 h. Supernatants were analysed for LDH release. (**C**) HPKs were stimulated with Biotin-labelled Poly(I:C) and LL37 complexes (1 μg/ml) for 18 h. Cell lysates were incubated with Streptavidin beads and immunoprecipitates were subjected to SDS-PAGE followed by immunoblotting with indicated antibodies. (**D, E**) Cell lysates from (**D**, *upper panel*) *RIG-I-*deficient (sgRIG-I) or (**E**, *lower panel*) *MDA5*-deficient N/TERT-1 cell-lines (sgMDA5) treated with LL37/Poly(I:C) complexes for 18 h or medium (-) were subjected to SDS-PAGE and immunoblotting with indicated antibodies. (**F, G**) HEK293^IL-36γ^ cells were transfected with empty vector (-) or GSDME with and without MAVS for 24 h and (**F**) cell lysates were subjected to SDS-PAGE and immunoblotting with indicated antibodies or (**G**) supernatants were measured for LDH release. Data are presented as a representative (**A,** **C, F**) of three independent experiments or are presented mean ±S.E.M. of 3 independent experiments and subjected to a (**B, D, E**) two-way ANOVA followed by Šidák’s or (**G**) one-way ANOVA followed by Tukey’s multiple comparisons test. *p<0.05, **p<0.01, ****p<0.0001. ns = non-significant. Lipo = Lipofectamine 2000, M = protein marker, sg = single-guide RNA.
