# Supplementary material for: LL37 complexed to double-stranded RNA induces RIG-I-like receptor signalling and Gasdermin E activation facilitating IL-36γ release from keratinocytes
Source: Cell Death Dis. 2025 Mar 22;16(1):198. doi: 10.1038/s41419-025-07537-9 (PMC11929817; doi:10.1038/s41419-025-07537-9)

Uncropped WBs:
Main figures

Figure 1 A

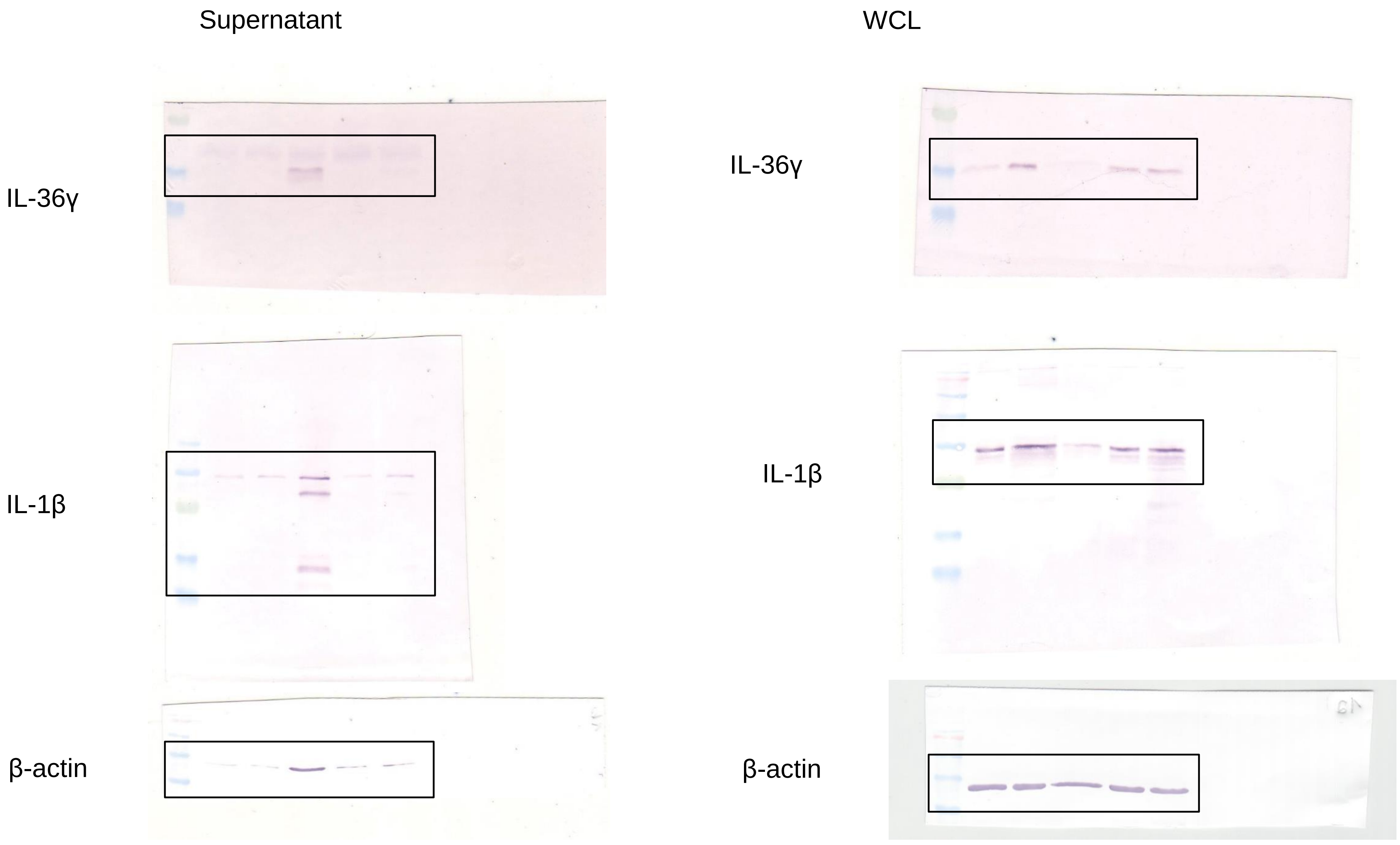

Figure 1 D

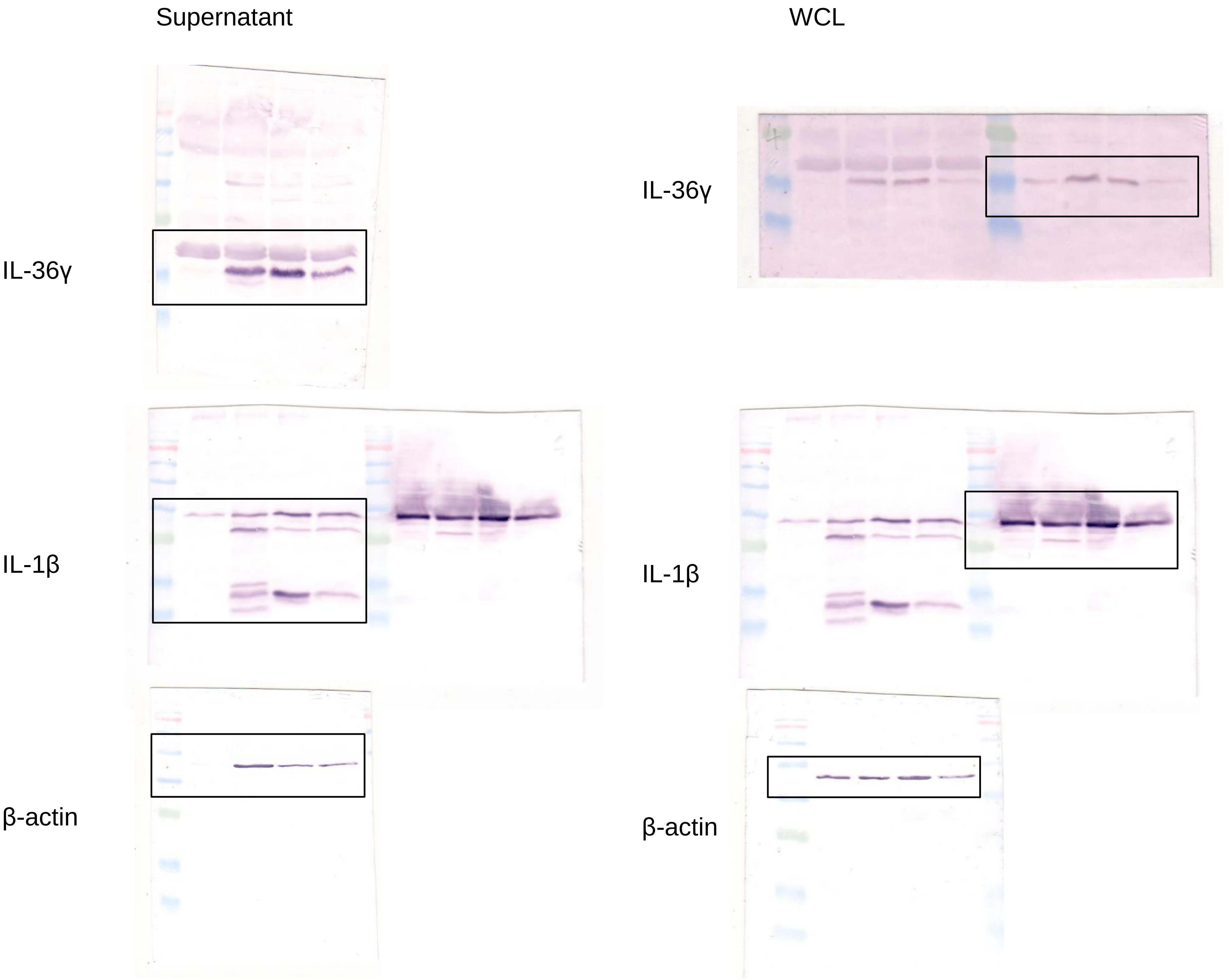

Figure 1 F

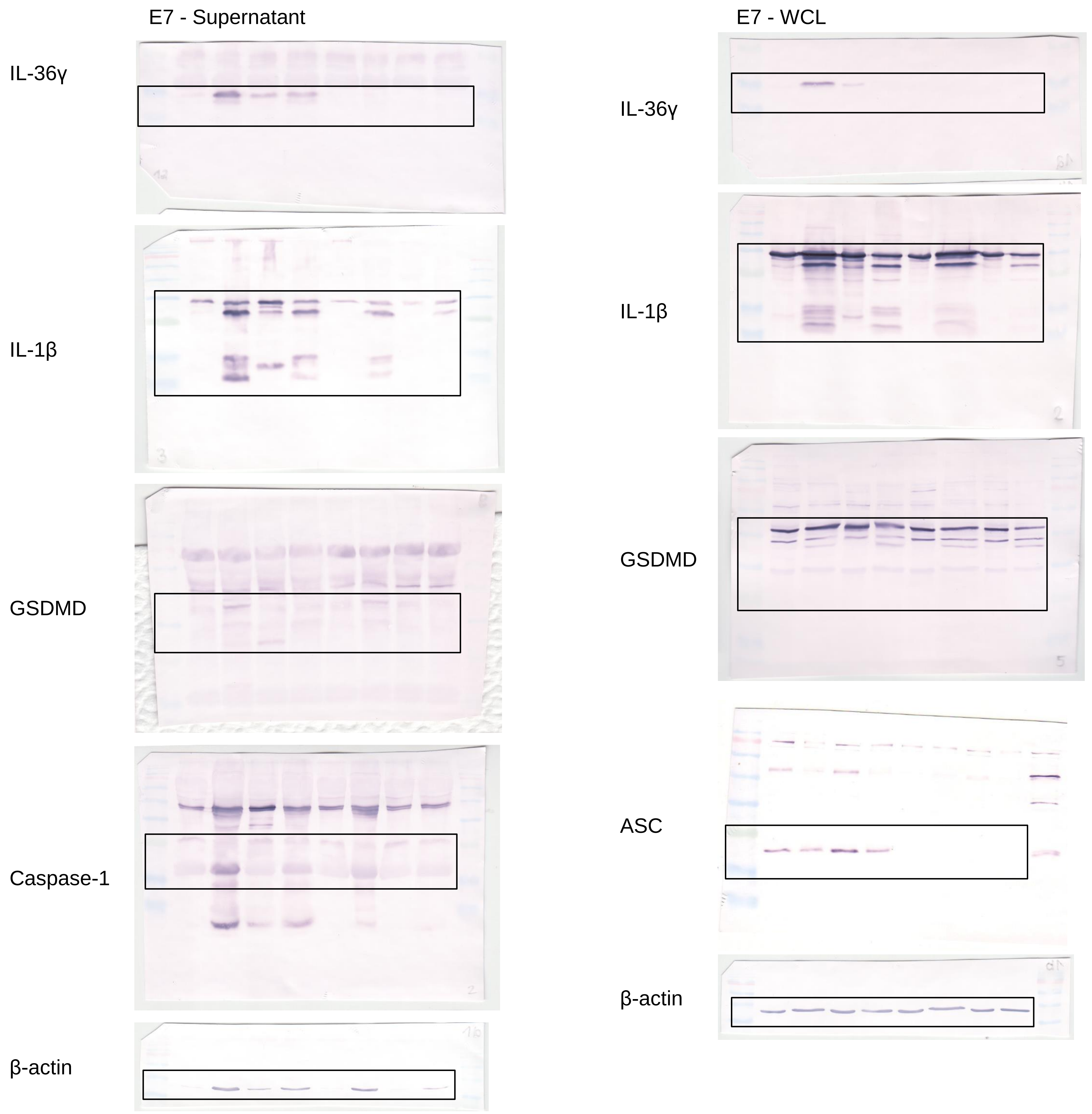

Figure 1 G

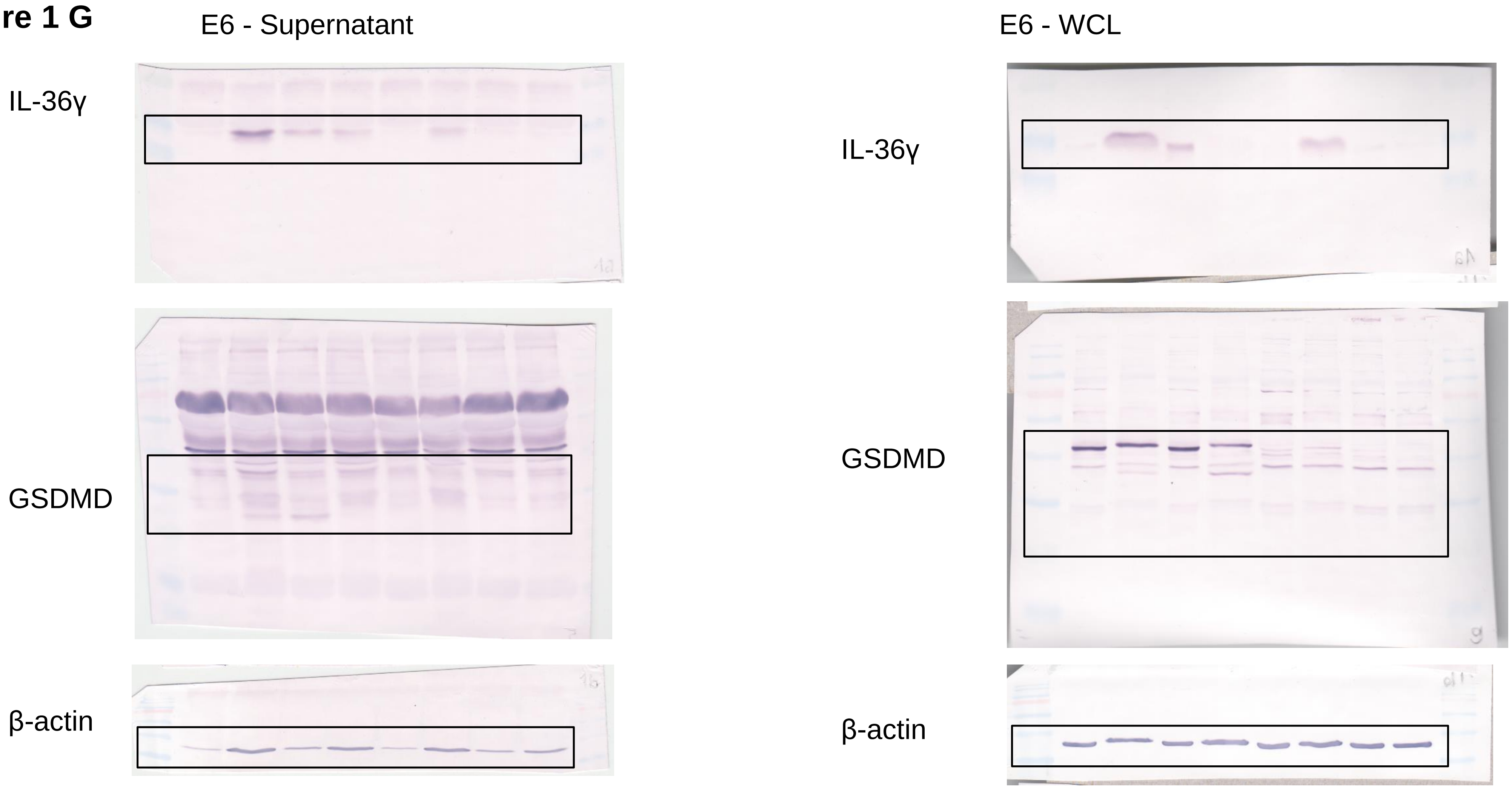

Figure 2 A

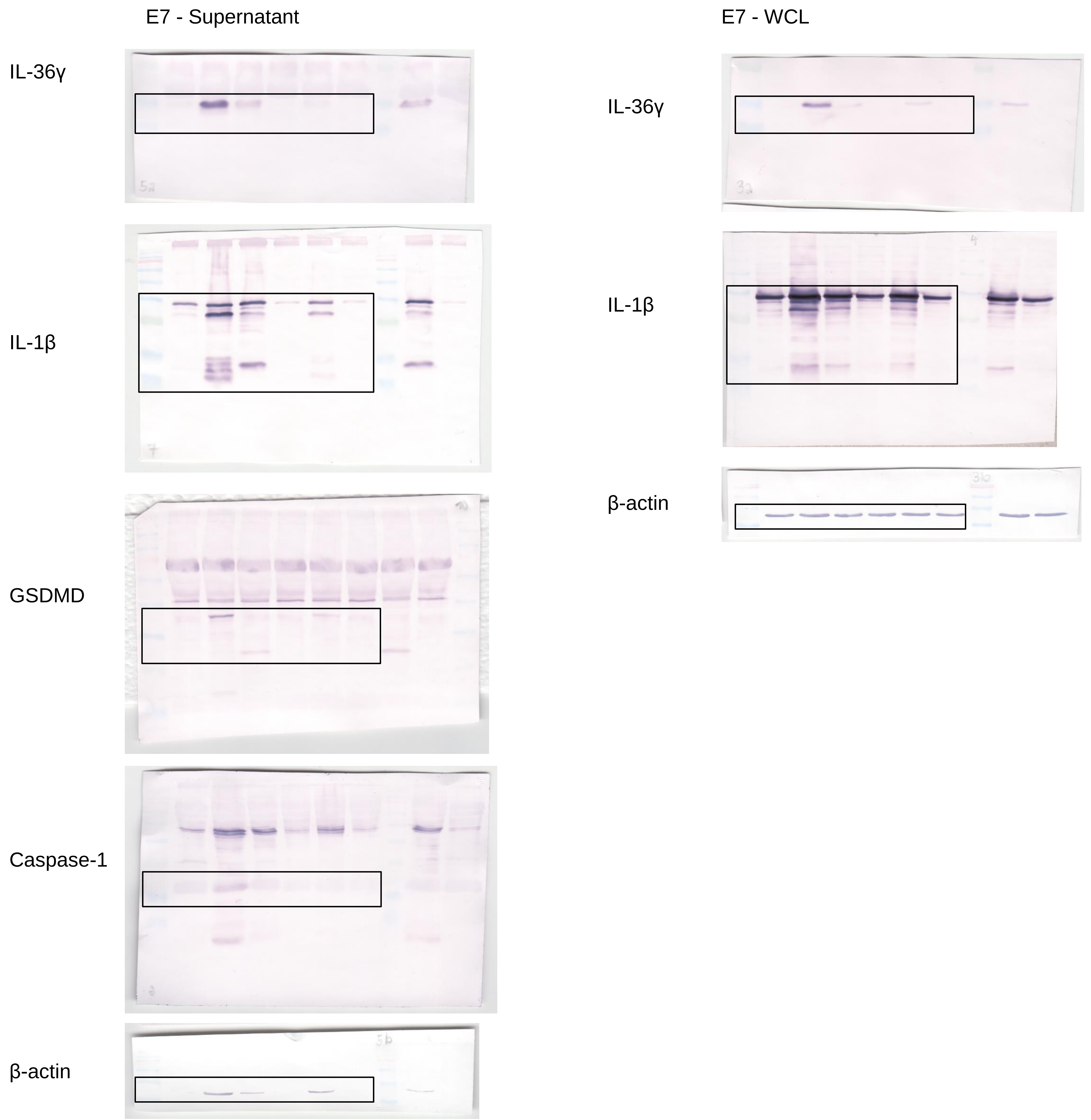

Figure 2 D

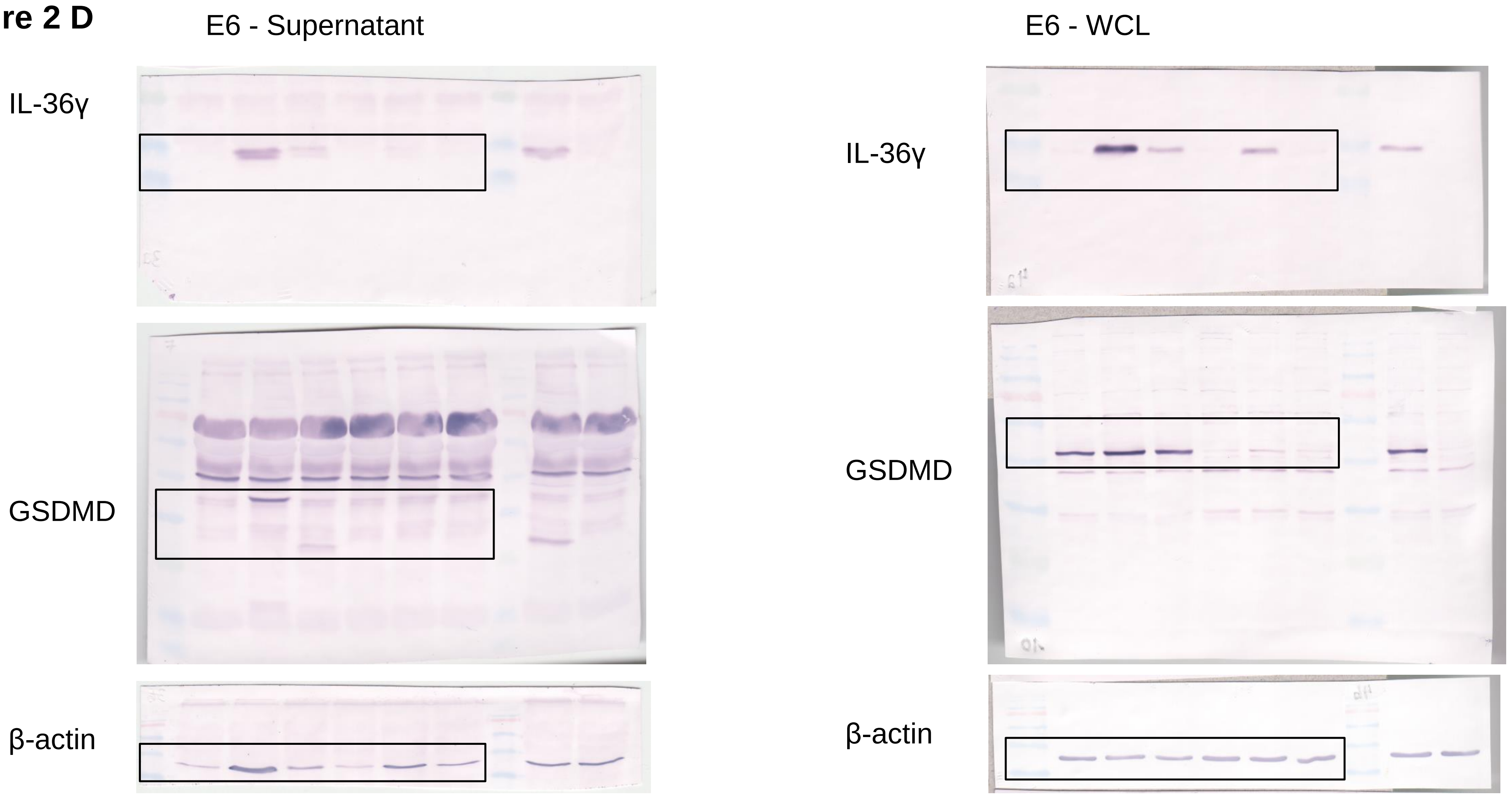

Figure 3 E

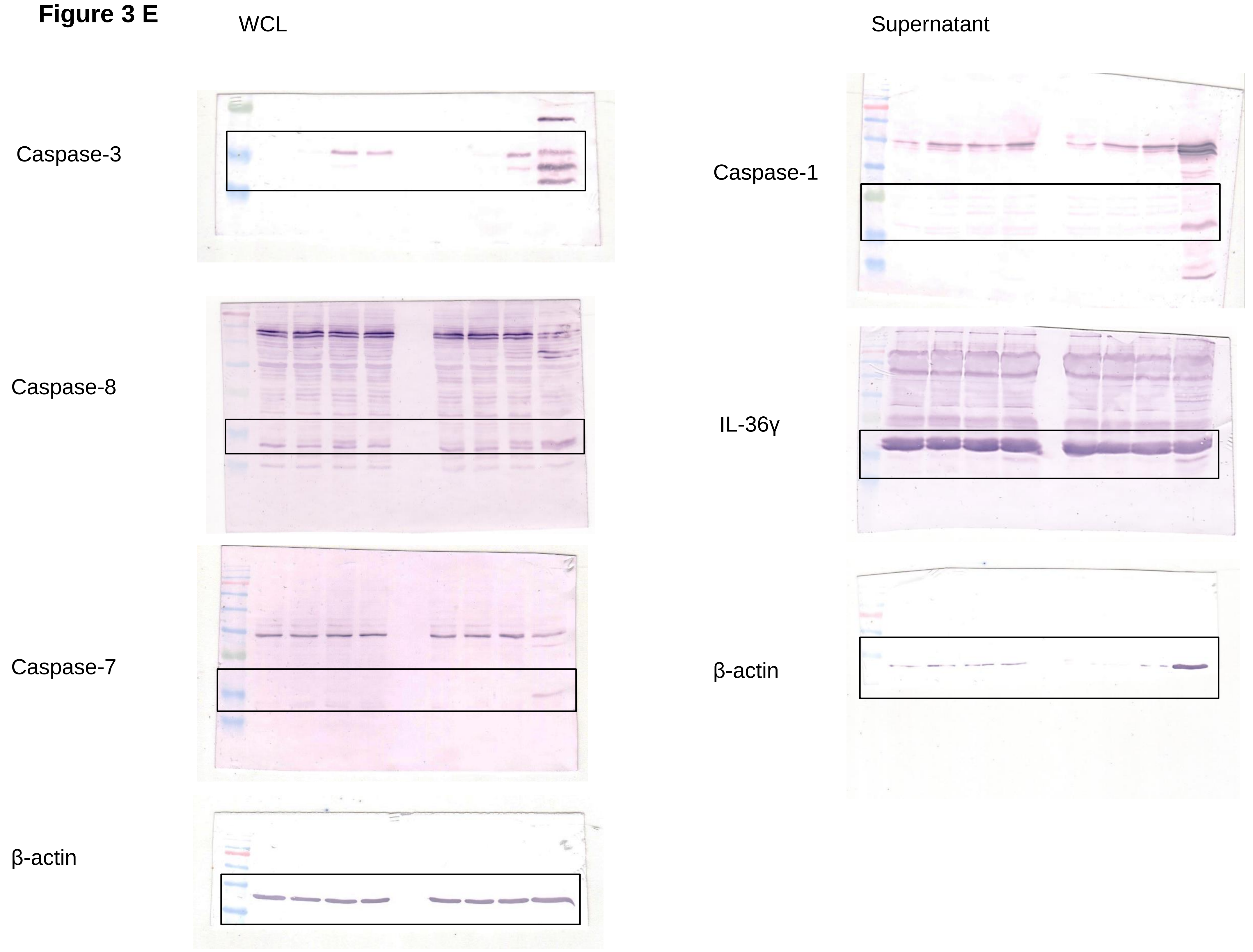

Figure 3 F

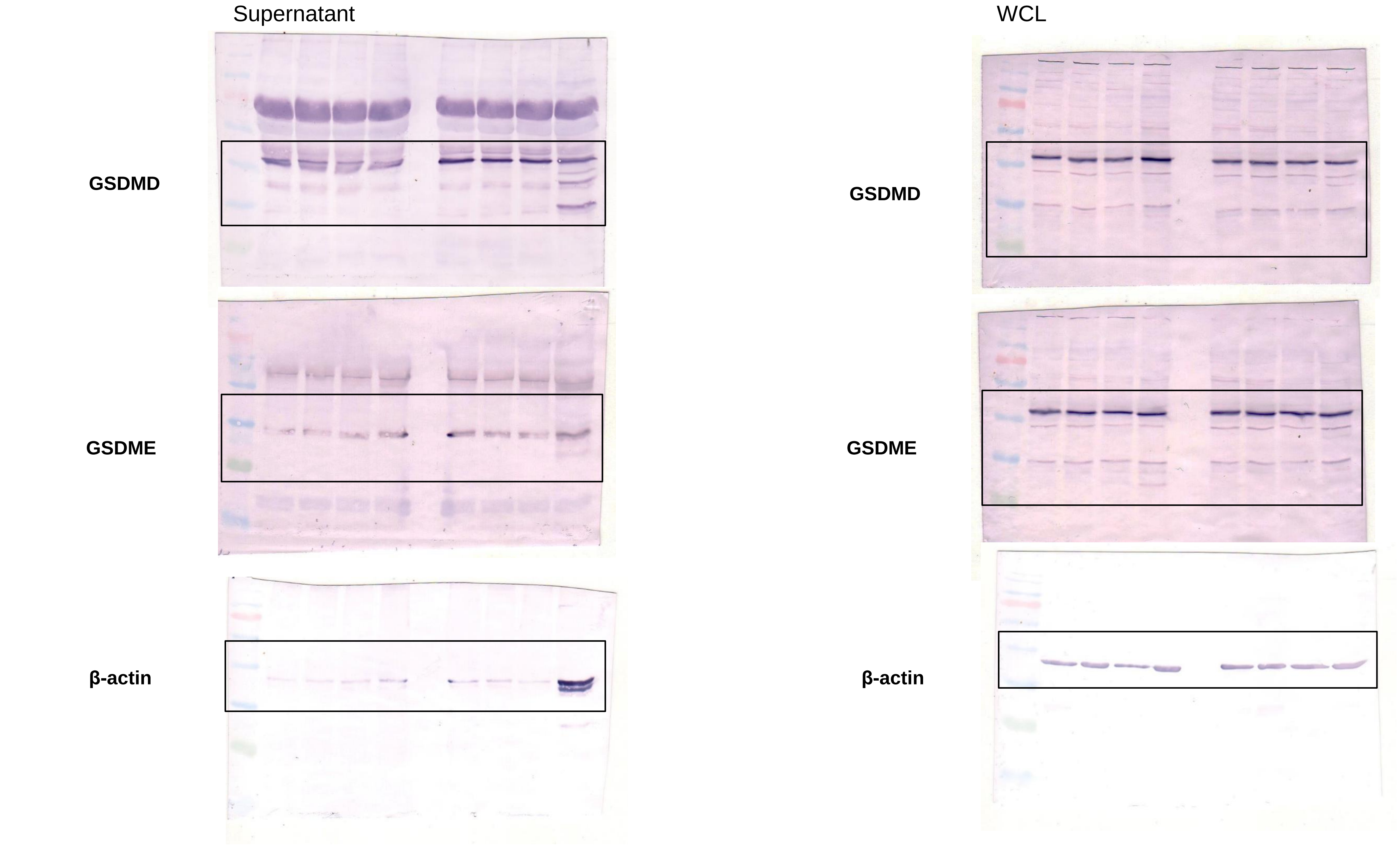

Figure 4 A

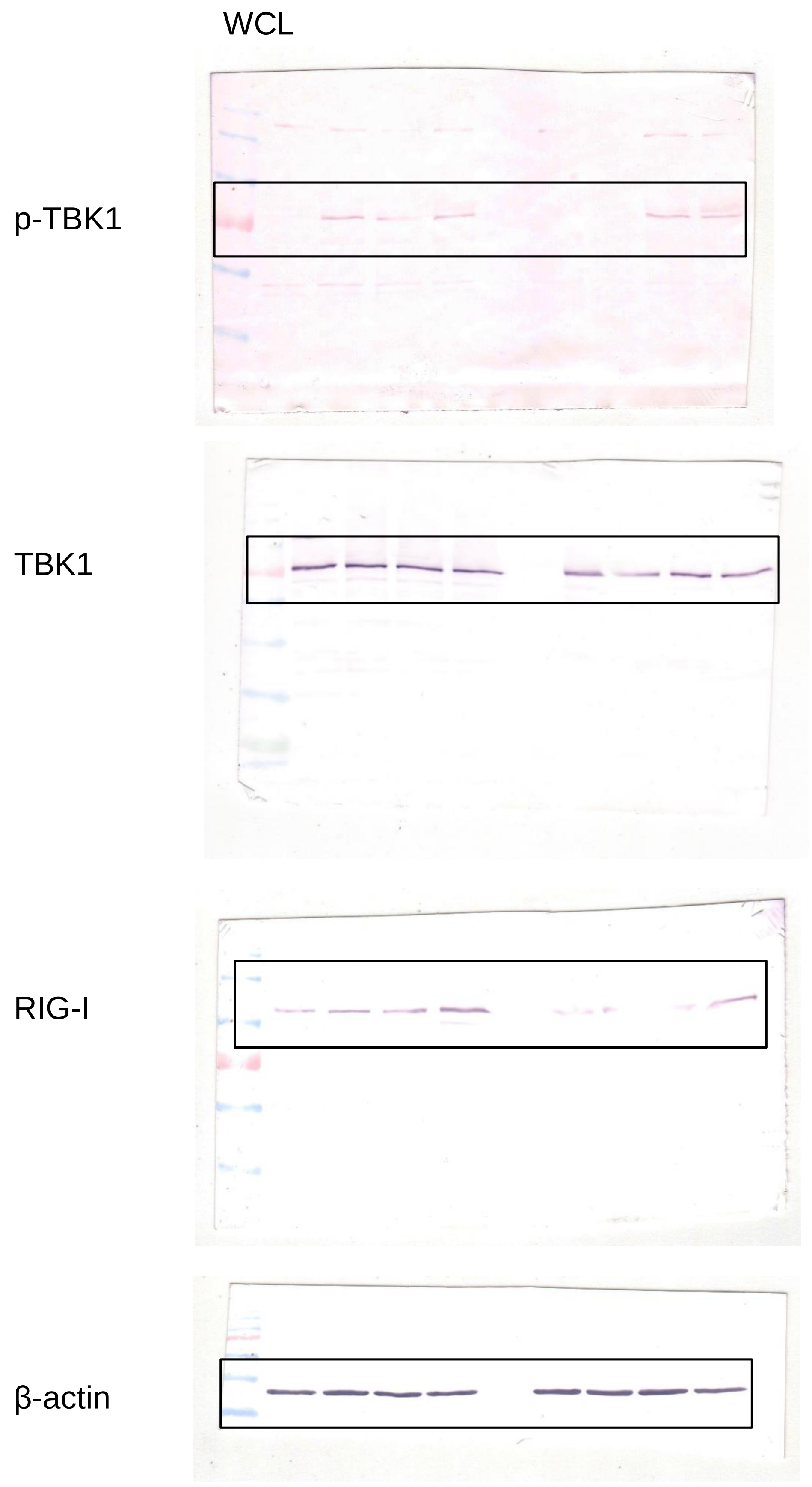

Figure 4 B

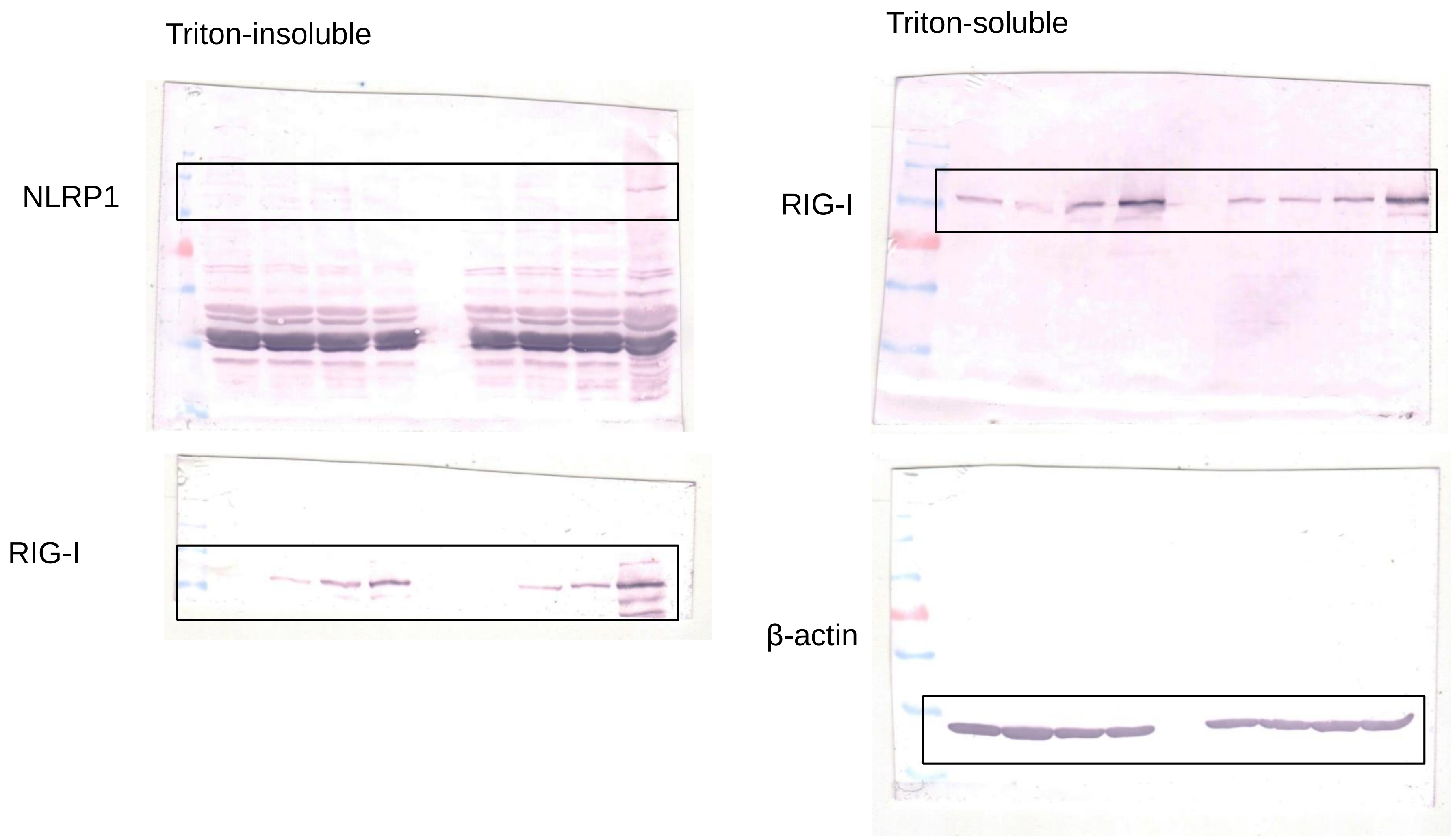

Figure 4 C

WCL

IL-36γ

RIG-I

MDA5

MAVS

NLRP1

β-actin

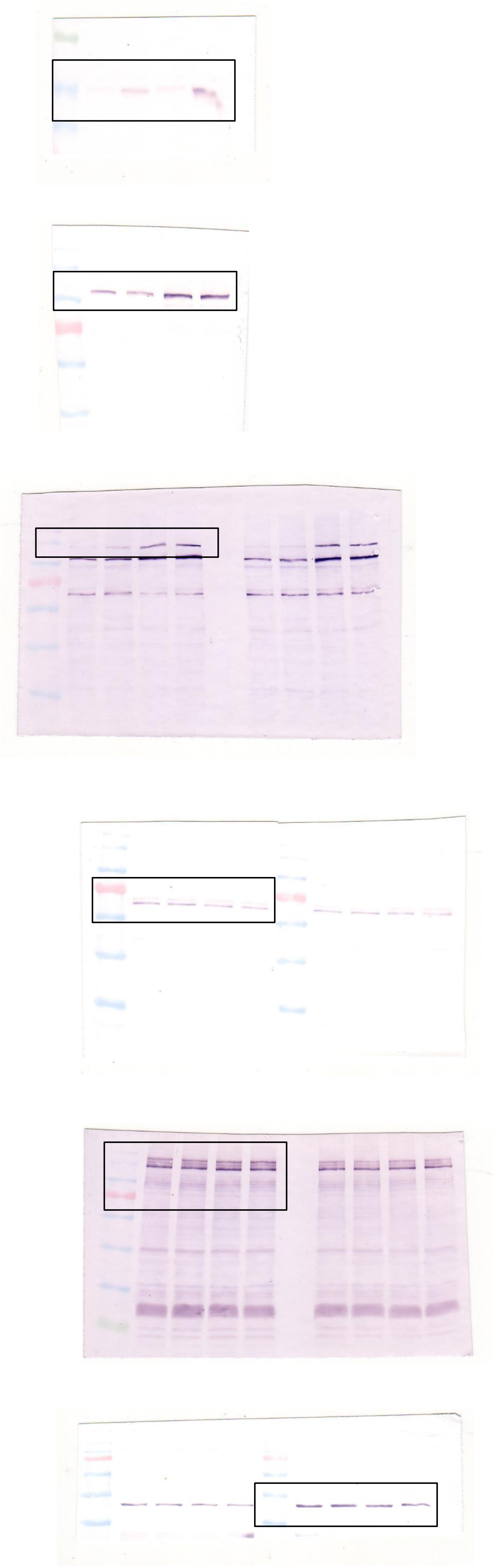

Figure 5 D

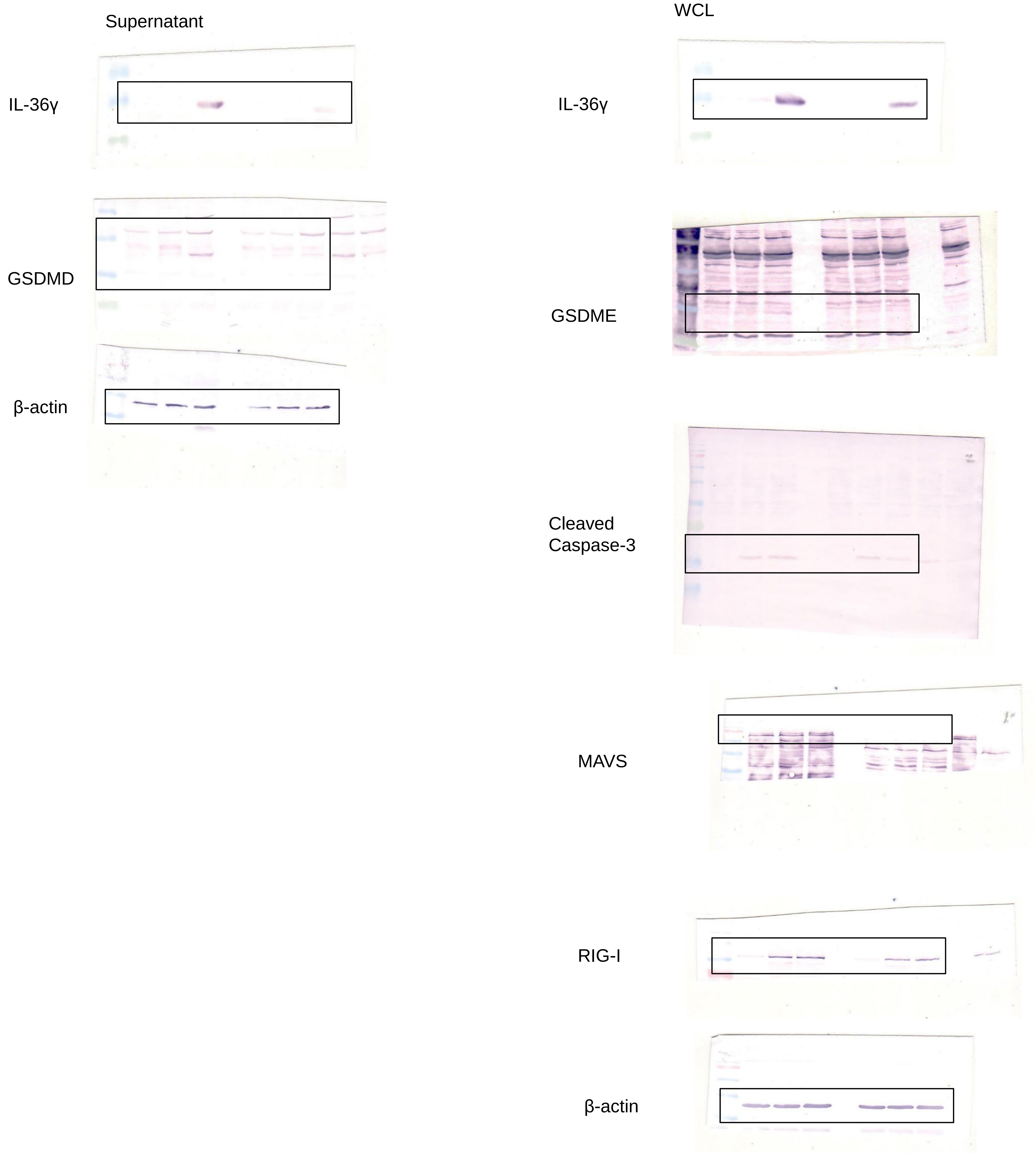
Supernatant
WCL
IL-36γ
GSDMD
β-actin
IL-36γ
GSDME
Cleaved
Caspase-3
MAVS
RIG-I
β-actin

Figure 5 E

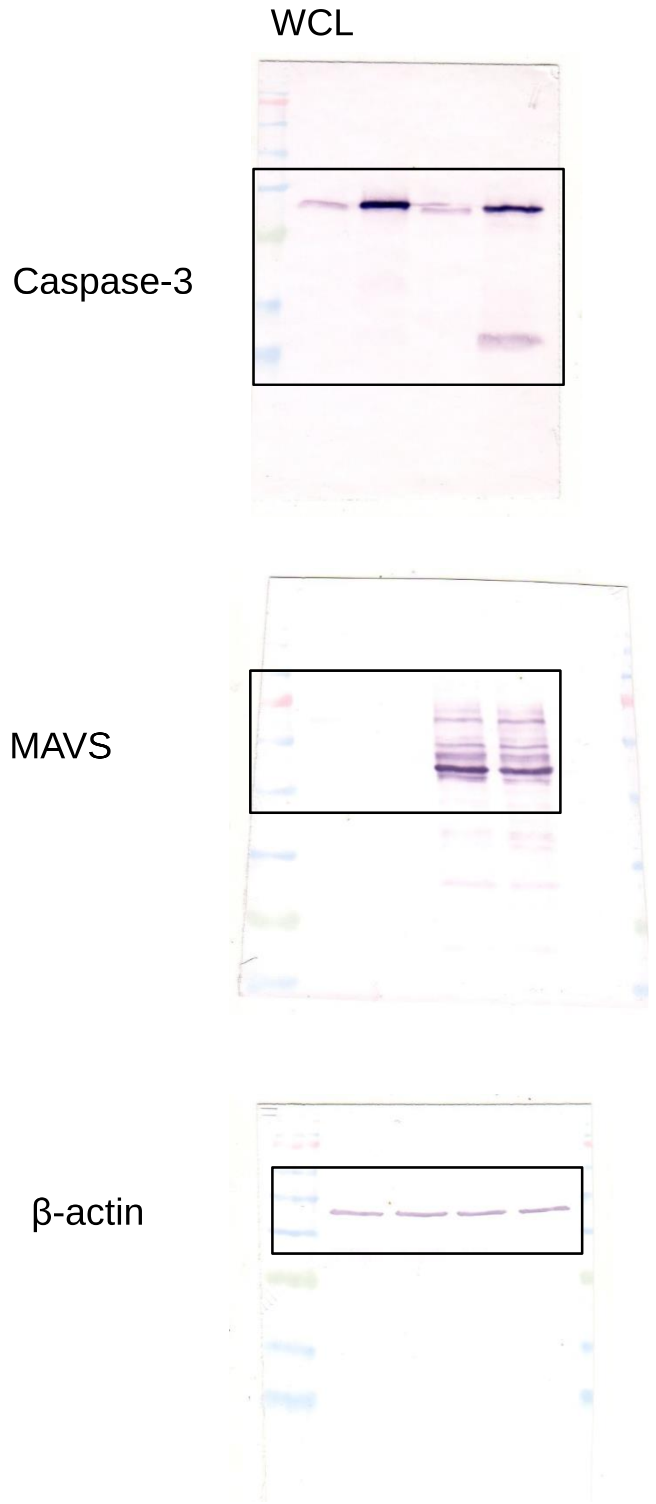

Figure 5 F

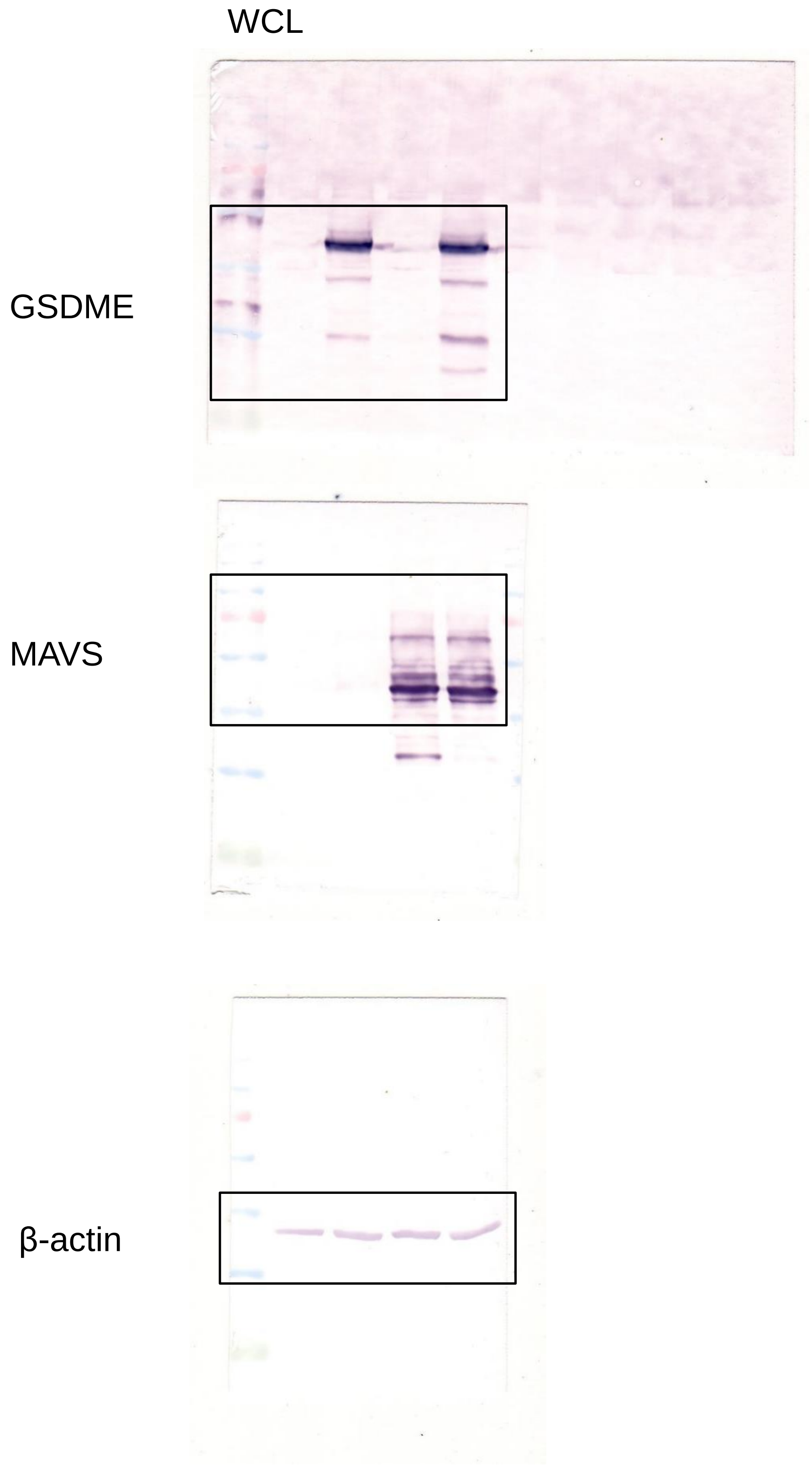

Supplementary Figures

Figure 1 B

Supernatant

IL-36γ

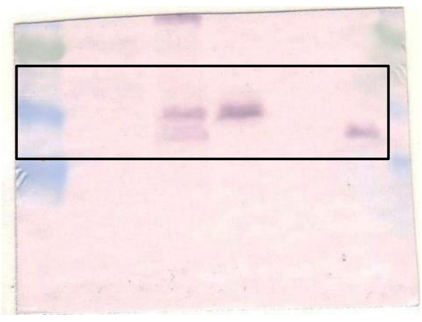

β-actin

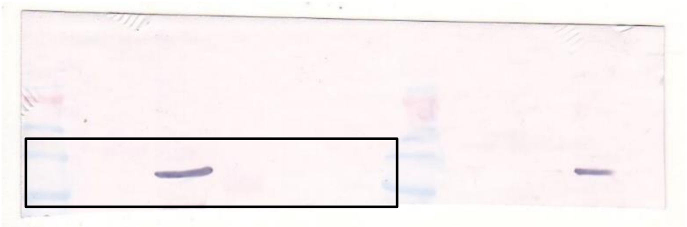

Figure 1 F

Caspase-3

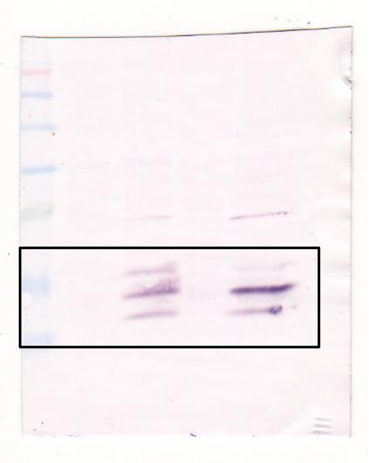

Caspase-7

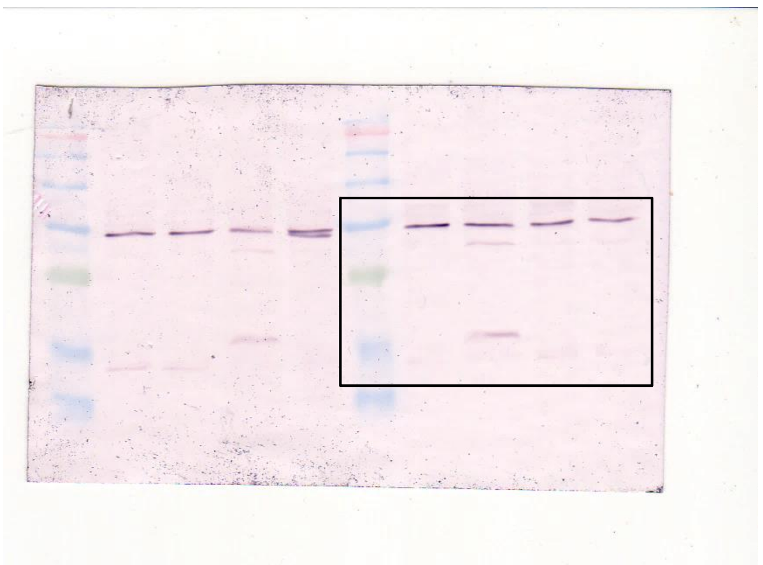

Caspase-8

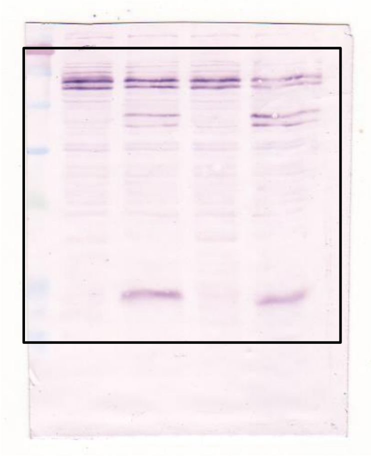

β-actin

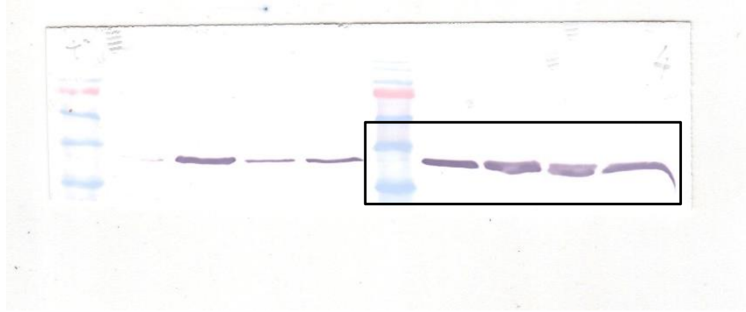

Figure 1 G

Supernatant

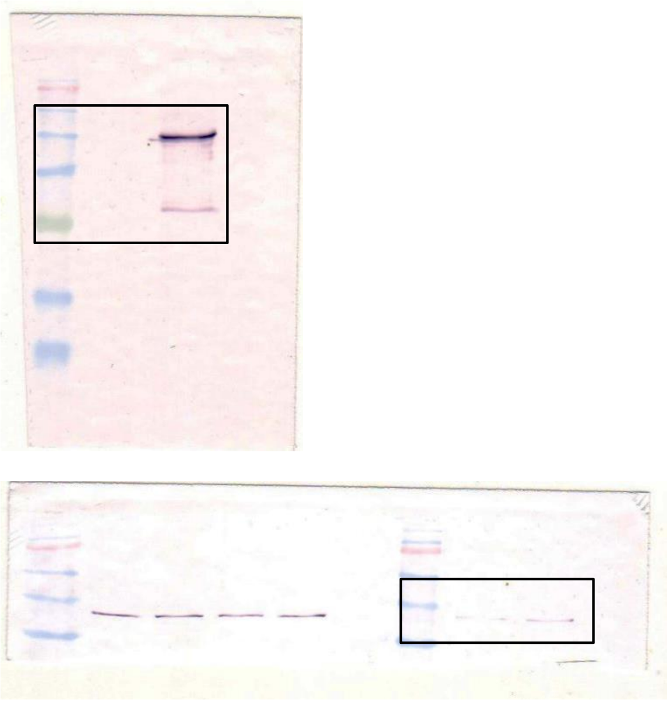

GSDMD

β-actin

Figure 1 H

Supernatant

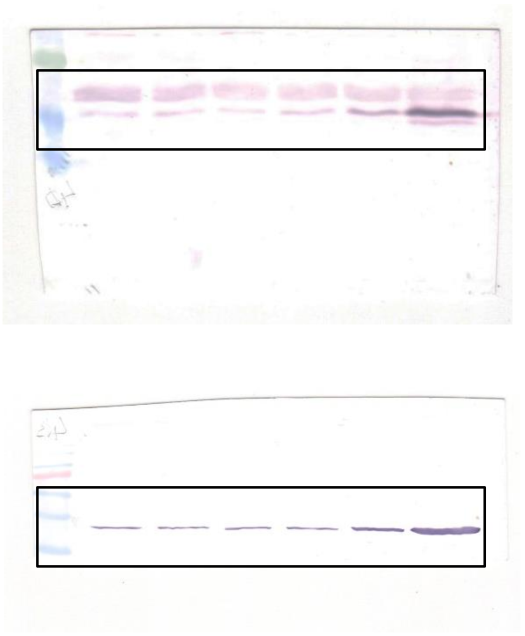

IL-36γ

β-actin

Figure 2 I

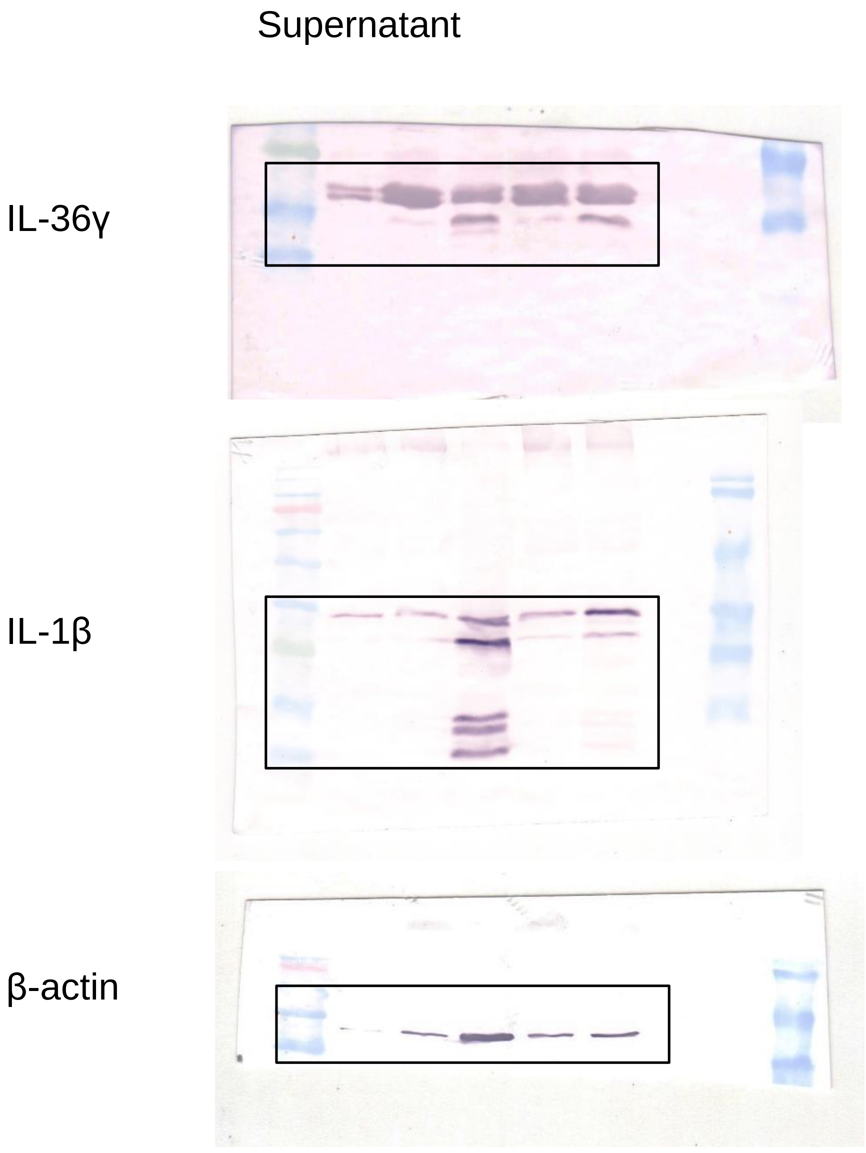

Figure 2 J

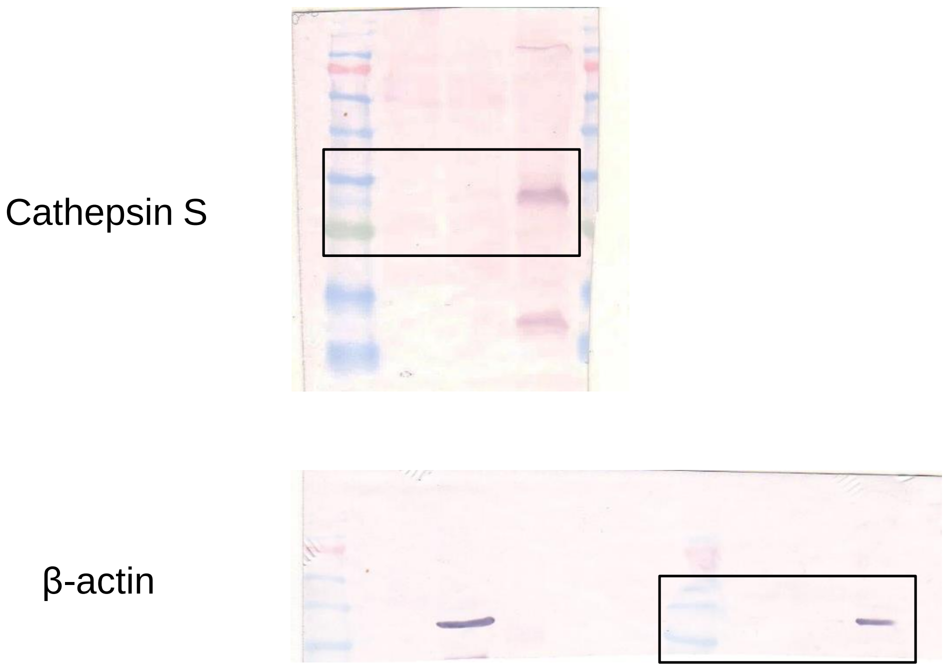

Figure 3 A

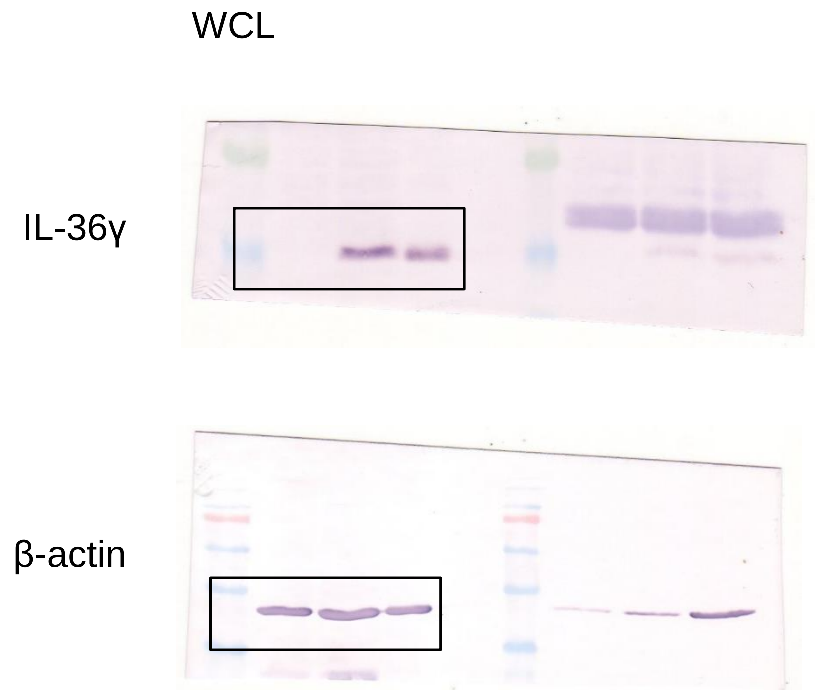

Figure 3 D

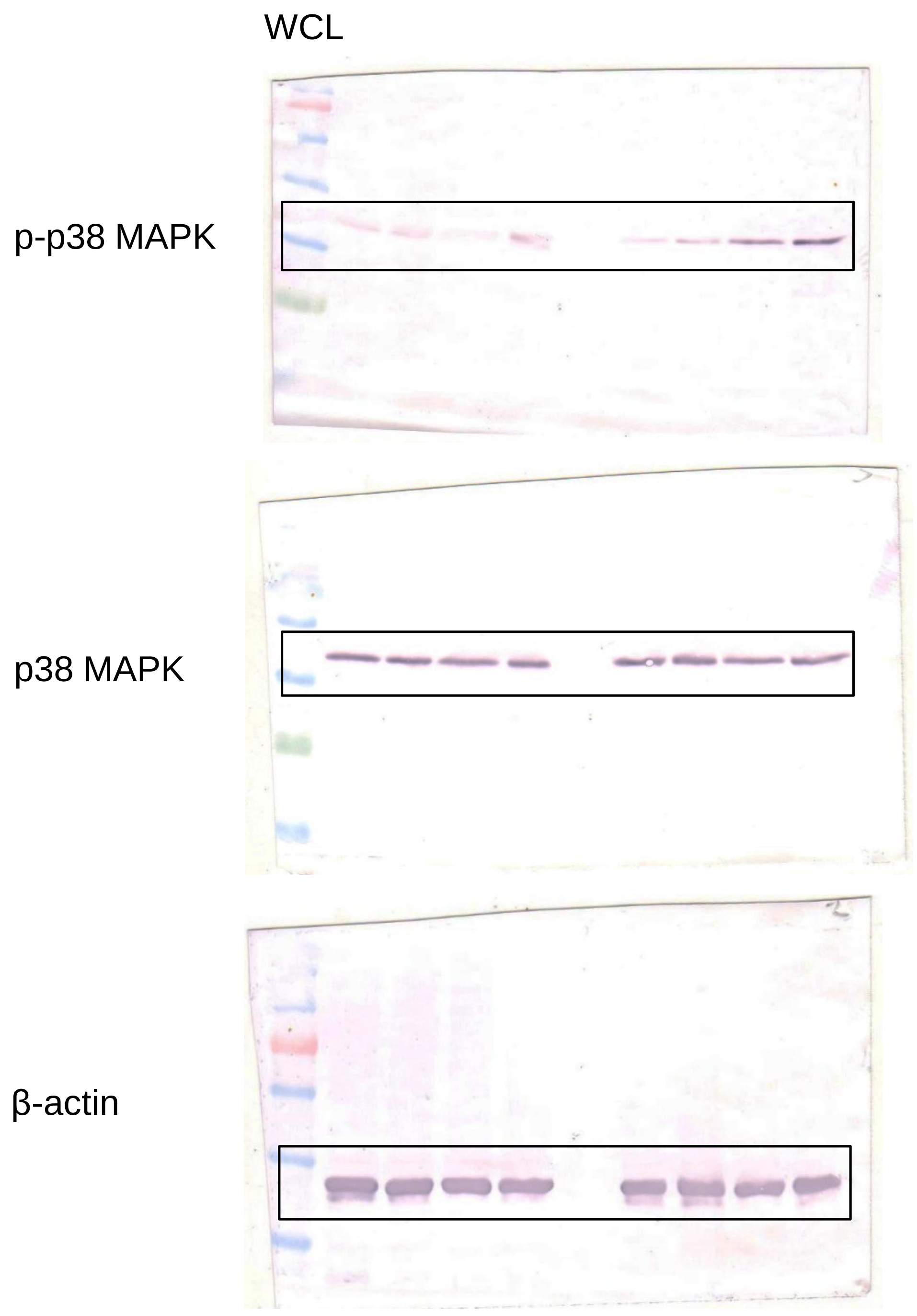

Figure 4 A

GSDME

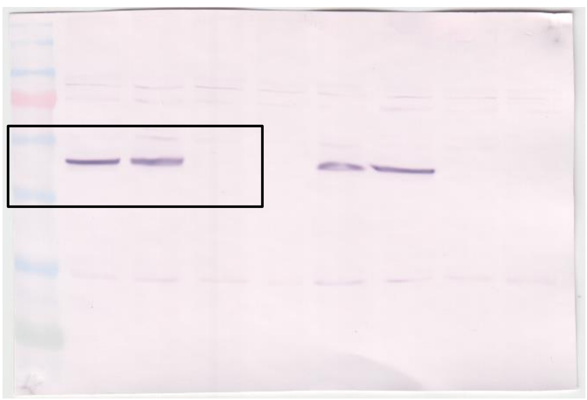

GSDMD

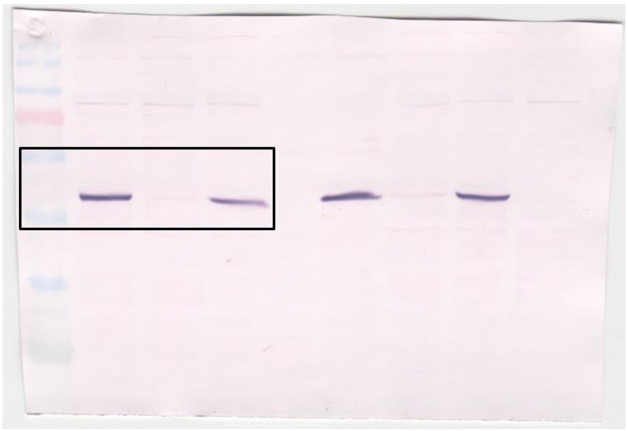

β-actin

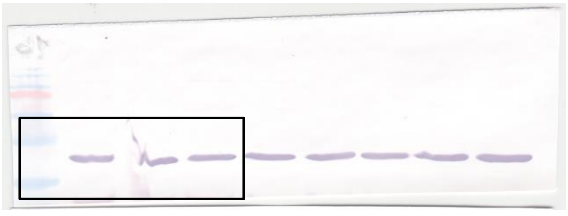

Figure 6 A

WCL

MAVS

β-actin

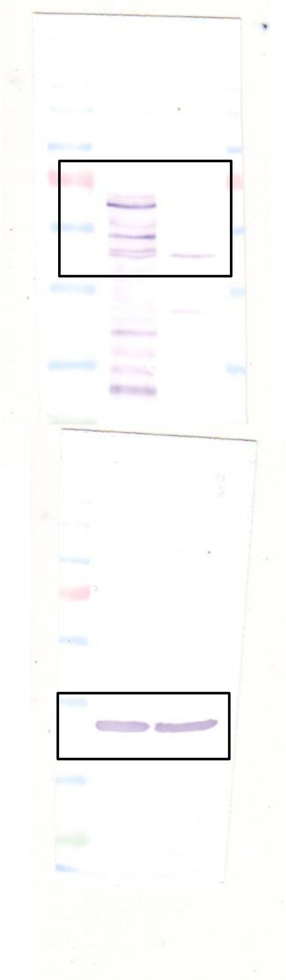

Figure 6 C

IP

WCL

RIG-I

NLRP1

β-actin

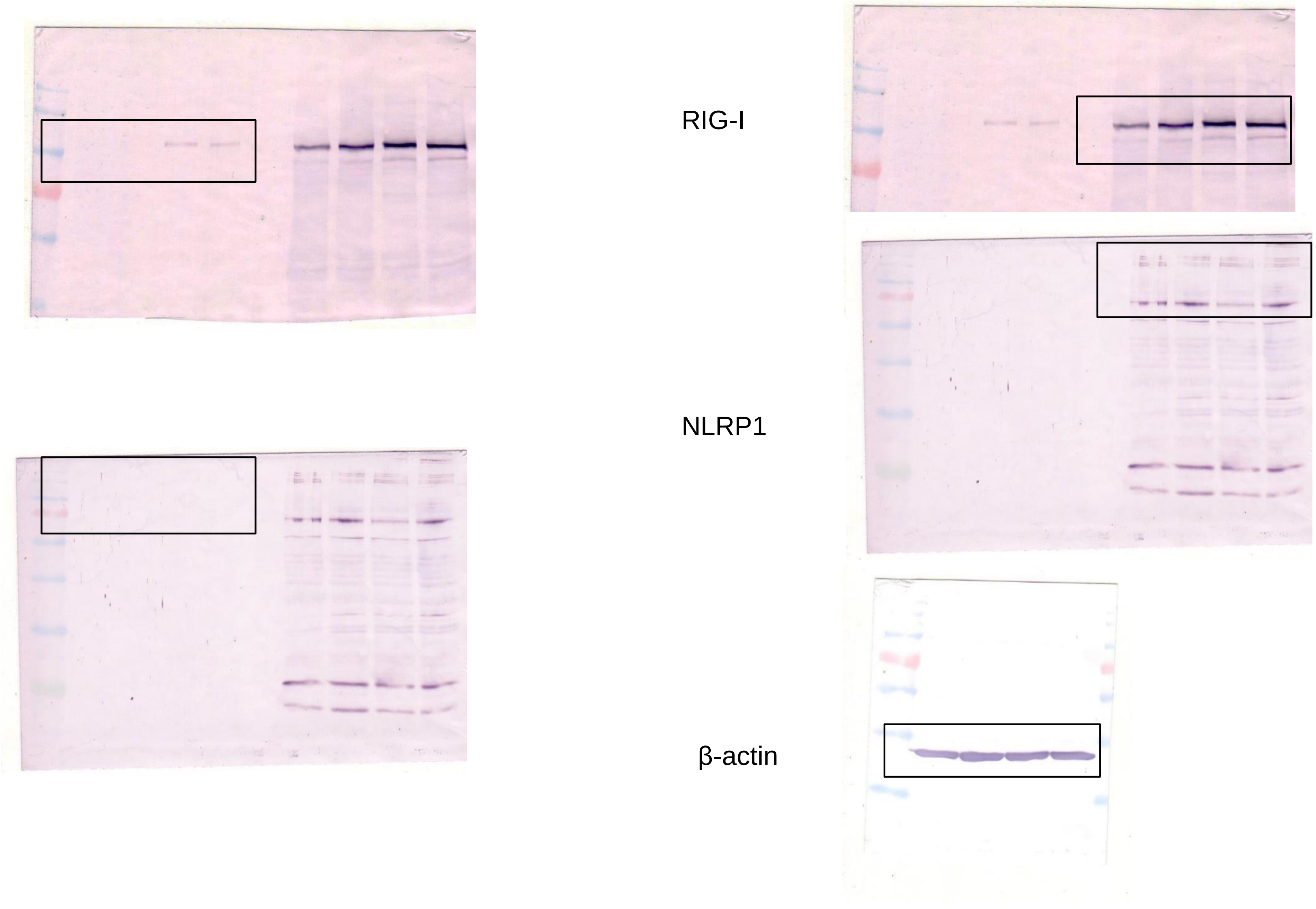

Figure 6 D

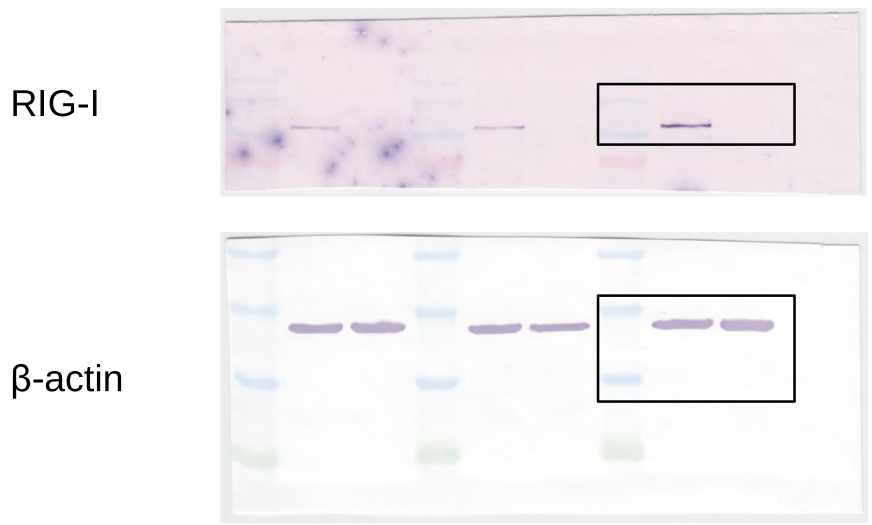

Figure 6 E

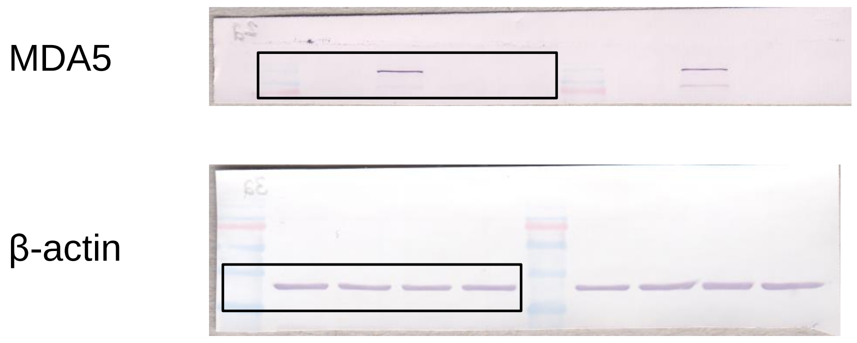

Figure 6 F

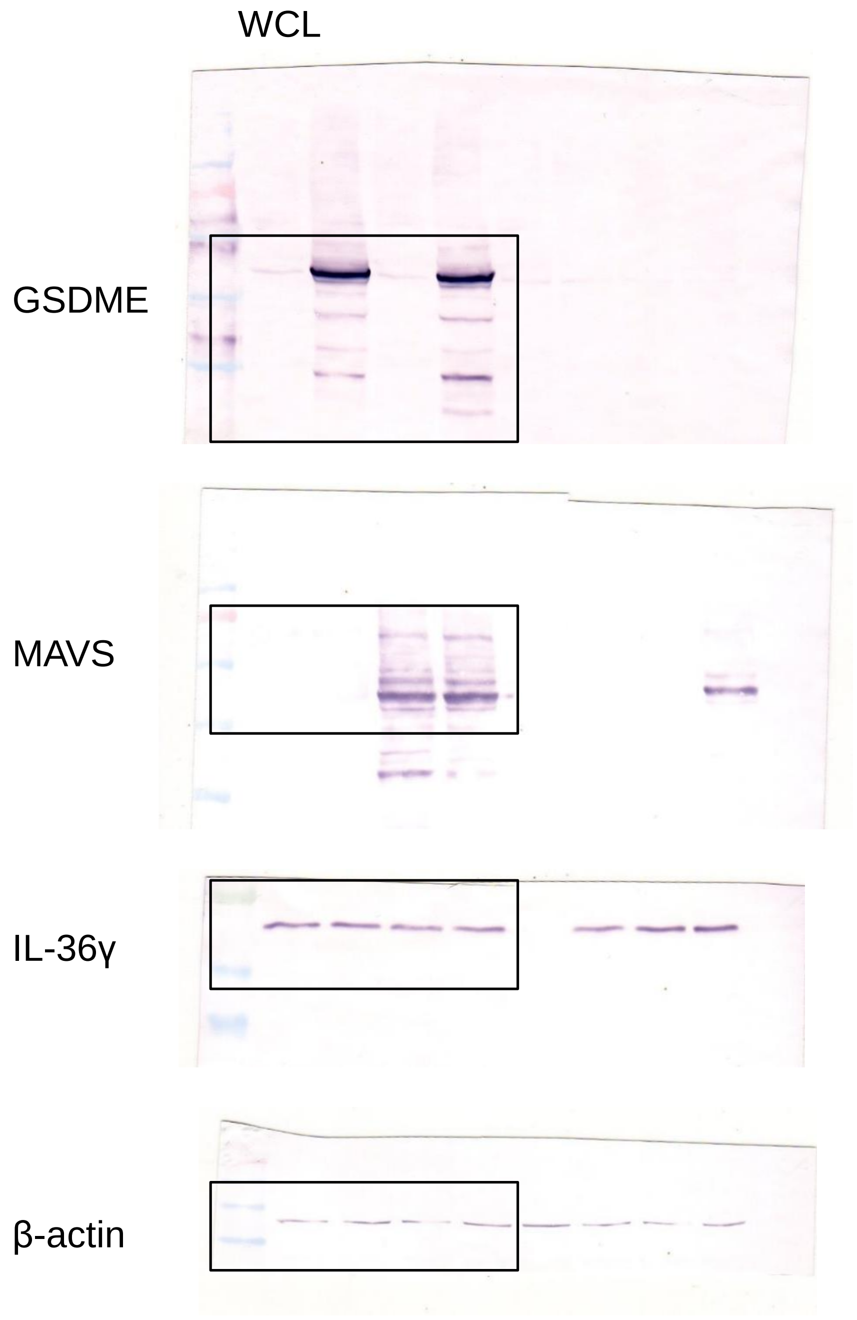

Figure 6 F

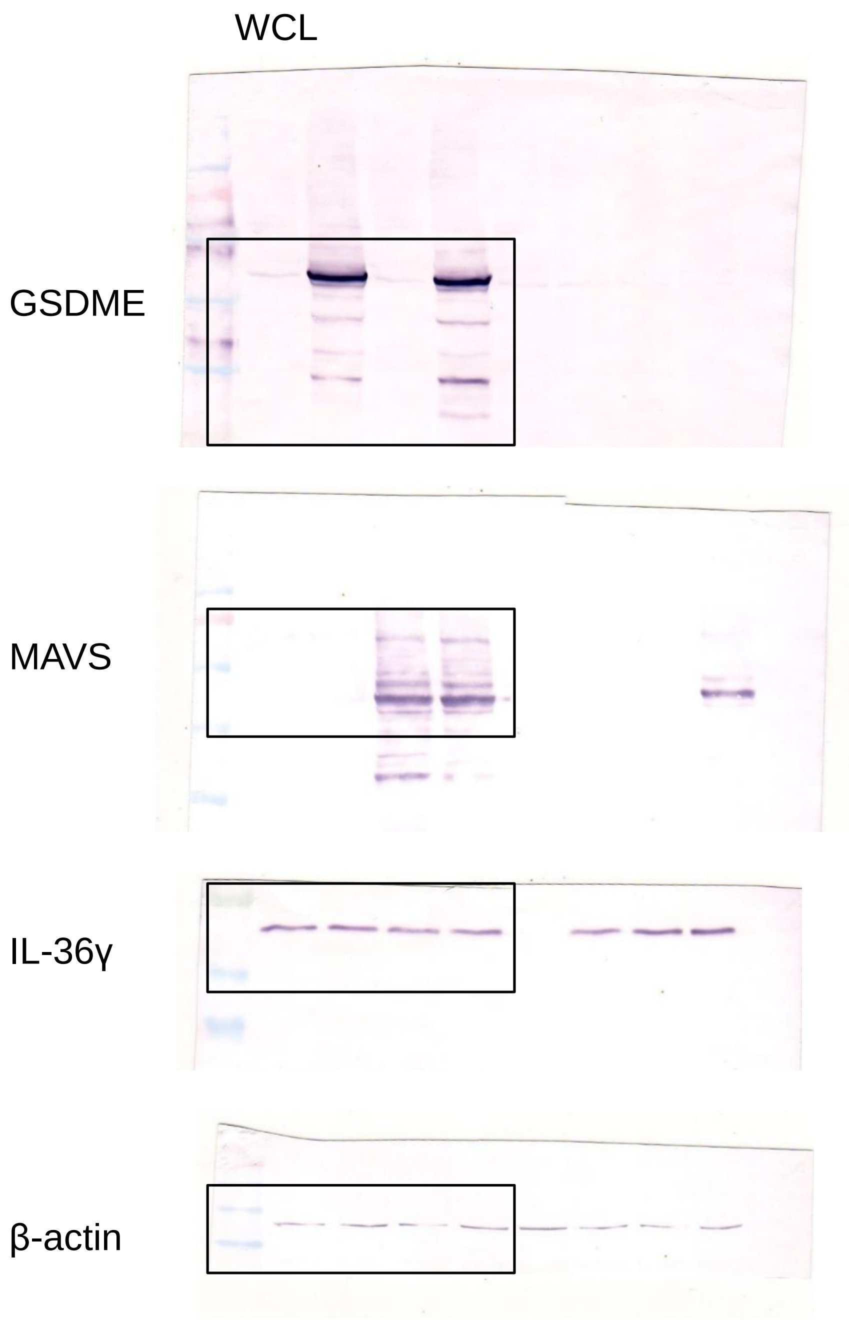

Supplement: Supplementary file 2 — Uncropped blots [file 41419_2025_7537_MOESM2_ESM.pdf]
